# Subtype-dependent PD-L1 stability and immune context shape immunotherapy response in hepatocellular carcinoma

**DOI:** 10.3389/fimmu.2026.1822947

**Published:** 2026-05-01

**Authors:** Luca Grisetti, Clarissa J. C. Garcia, Paola Tarchi, Claudio Tiribelli, Devis Pascut

**Affiliations:** 1 National Institute of Gastroenterology—IRCCS “Saverio de Bellis”, Castellana Grotte, Italy; 2 Liver Cancer Unit, Fondazione Italiana Fegato, Trieste, Italy; 3 Department of Life Sciences, Università degli Studi di Trieste, Trieste, Italy; 4 Surgical Clinic, Azienda Sanitaria Universitaria Giuliano Isontina (ASUGI), Trieste, Italy

**Keywords:** atezolizumab, durvalumab, hepatocellular carcinoma (HCC), IFN-γ, immune checkpoint inhibitors (ICIs), molecular heterogeneity, PD-L1, tumor immune microenvironment

## Abstract

**Introduction:**

Immune checkpoint inhibitors (ICIs) targeting the PD-1/PD-L1 axis have expanded treatment options for hepatocellular carcinoma (HCC), yet only a subset of patients achieves durable responses. Limited efficacy reflects gaps in understanding PD-L1 regulation, including its biochemical heterogeneity, subtype-specific stability, and effects on tumor–immune interactions. We aimed to dissect these mechanisms and identify integrated biomarkers predictive of immunotherapy response.

**Methods:**

Human HCC and paired non-tumoral tissues, an HBV-driven transgenic mouse model, and five HCC cell lines representing Hoshida/Caruso subtypes were analyzed. PD-L1 expression, glycosylation, and stability were assessed by Western blot, flow cytometry, and immunohistochemistry. Functional assays included co-cultures of HCC cells with primary activated PBMCs from multiple healthy donors. Cells were treated with durvalumab or atezolizumab, in the presence or absence of IFN-γ stimulation. PD-L1 turnover was evaluated using cycloheximide and proteasome inhibition.

**Results:**

PD-L1 protein was increased in ~60% of tumors, particularly in virally driven HCC. Elevated PD-L1 in non-tumoral liver was associated with higher recurrence risk. Biochemical profiling revealed multiple PD-L1 species: mature N-glycosylated and intermediate forms indicative of enhanced stability were enriched in tumors. Glycosylated PD-L1 displayed prolonged half-life in S1/CL3 subtypes, whereas S2/CL1 cells exhibited rapid, proteasome-dependent turnover. PD-L1 abundance alone did not predict immune susceptibility: S1-like cells, despite higher PD-L1, were highly sensitive to CD8^+^ T-cell–mediated killing and PD-L1 blockade, whereas S2-like cells were more resistant to cytotoxicity. ICIs induced donor-dependent cytotoxicity, with variable responder profiles; durvalumab outperformed atezolizumab, consistent with reduced glycosylation dependence. IFN-γ priming enhanced PD-L1 expression and restored immune responsiveness in low-responder co-cultures. Overall, tumors with stable glycosylated PD-L1 (S1-like) rely on PD-L1–mediated immune suppression and may benefit from PD-L1–targeted therapies, whereas tumors with low or rapidly turned-over PD-L1 (S2-like) exhibit dynamic PD-L1 regulation with increased reliance on *de novo* synthesis, limiting responsiveness to PD-L1 blockade alone and supporting the need for combinatorial approaches.

**Conclusions:**

PD-L1 regulation in HCC depends on molecular subtype, post-translational stability, and host immune competence. Integrating these factors provides a framework for guiding patient selection and optimizing immunotherapy.

## 1 Introduction

Hepatocellular carcinoma (HCC) is the third leading cause of cancer-related mortality worldwide, with most patients diagnosed at advanced stages when prognosis is poor (1, 2). Immune checkpoint inhibitors (ICIs) have transformed the therapeutic landscape of advanced HCC, yet clinical benefit is heterogeneous (3). The IMbrave150 trial in 2020 led to the approval of atezolizumab (anti–PD-L1) and bevacizumab (anti–vascular endothelial growth factor [VEGF]) combination as the first-line standard of care with improved overall survival (OS) *versus* sorafenib (median 19.2 *vs.* 13.2 months) (4). Despite recent advances, durable responses occur in only ~30% of patients, and the molecular determinants distinguishing responders from non-responders remain poorly understood (4).

Programmed death-ligand 1 (PD-L1) has long been explored as a predictive biomarker, but its clinical utility in HCC remains inconsistent (5). This inconsistency reflects an apparent paradox: while PD-L1 suppresses T-cell activation, tumors with higher PD-L1 often respond better to programmed death-1 (PD-1)/PD-L1 blockade (6). Recent single-cell transcriptomic analyses of patients treated with atezolizumab plus bevacizumab indicate that pre-existing immune infiltration, rather than PD-L1 abundance alone, drives response (7). Static measurements of PD-L1 thus fail to capture the functional context of checkpoint blockade.

One explanation is that PD-L1 is biochemically heterogeneous and undergoes extensive post-translational modification, with N-linked glycosylation influencing its stability, subcellular localization, and PD-1 engagement (8). Glycosylation prolongs PD-L1 half-life (9) and affects antibody recognition, with atezolizumab partially dependent on specific glycoforms, whereas durvalumab is largely glycosylation-independent (10, 11). Despite these implications, the biochemical diversity of PD-L1 in HCC and its impact on therapeutic response remain poorly defined.

HCC’s molecular heterogeneity further complicates interpretation. Transcriptomic analyses classify HCC into three major subtypes (S1, S2, and S3) with distinct biology and prognosis (12). S1 tumors show transforming growth factor-beta (TGF-β)–activated WNT signaling (13), stromal infiltration (14), and cholangiocarcinoma-like gene signature (15). S2 tumors are proliferative and stem-like, with elevated alpha-fetoprotein (AFP) (13, 16), epithelial cell adhesion molecule (EPCAM) (17), glypican-3 (GPC3) (18), and MYC proto-oncogene (MYC)/protein kinase B (AKT) activation (19). S3 tumors are well-differentiated and metabolically enriched (13). A complementary CL1–CL3 classification proposed by Caruso and collaborators captures a mesenchymal-to-epithelial gradient across HCC (20). S1 and S2 subtypes are linked to poor prognosis, whereas S3 tumors generally have better outcomes (12). HCC cell lines recapitulate S1/S2 features and display distinct immune-evasion strategies (21), yet it remains unknown whether these subtypes drive different mechanisms of PD-L1 regulation, particularly posttranslationally, and how this affects PD-1/PD-L1 dependence (22).

Tumor-intrinsic features alone cannot explain immunotherapy outcomes. Effective checkpoint blockade requires both a susceptible tumor and a competent host immune system (23). Inter-individual variation in immune competence is substantial (24), yet most biomarker efforts in HCC remain tumor-centric, with limited integration of host immune competence and its interaction with tumor PD-L1 biology.

To address these gaps, we integrated patient-derived HCC tissues, an HBV-driven murine model, and molecularly defined HCC cell lines to study PD-L1 expression, biochemical heterogeneity, and subtype-specific regulatory dynamics. By resolving PD-L1 biochemical diversity, mapping subtype-dependent regulatory programs, and examining how PD-L1 stability intersects with intrinsic immune cytotoxic potential, we identify determinants of immunotherapy sensitivity beyond conventional PD-L1 assessment. These findings provide a mechanistic framework for understanding why only a subset of HCCs benefit from PD-L1–targeted therapies and suggest novel avenues for biomarker development in immuno-oncology.

## 2 Materials and methods

### 2.1 Human samples

Fifty-nine consecutive patients diagnosed with HCC according to EASL criteria and undergoing partial hepatectomy at the University Hospital of Trieste (Italy) were enrolled (**Table 1**). Eligible participants had histologically confirmed HCC. Patients with concurrent hepatic or extrahepatic malignancies or pediatric cases were excluded. Tumor and paired adjacent non-tumoral tissues were collected following a standardized sampling protocol. Samples were preserved in RNAlater or snap-frozen at −80 °C, with paired portions formalin-fixed and paraffin-embedded (FFPE) for histological evaluation (ASUGI, Trieste).

**Table 1:** Clinical and pathological characteristics of HCC patients.

Variables	HCC (N = 59)
Gender (M/F)	45/14
Age	70.1 [62.4-75.0]
BMI	25.9 [23.6-30.2]
Hemoglobin (g/dl)	14.1 [13.3-15.3]
Platelet (x10^3^/mm^3^)	165 [112-222]
AST (U/l)	31 [24-53]
ALT (U/l)	28 [19-54]
Pseudocholinesterase (U/l)	6597 [5339-7452]
Total Bilirubin (mg/dl)	0.83 [0.58-1.02]
Direct Bilirubin (mg/dl)	0.19 [0.14-0.27]
Albumin (g/dl)	4.10 ± 0.47
INR	1.09 [1.04-1.14]
Creatinine (mg/dl)	0.82 [0.73-0.93]
Triglycerides (mg/dl)	108 ± 44
Cholesterol (mg/dl)	175 ± 37
HDL (mg/dl)	46 ± 12
AFP (ng/ml)	10.1 [3.7-136.1]
AFP classes (<20/20-40/>400ng/ml)	35/11/8
Etiology (HCV/HBV/MASLD or ALD/Mix)	18/6/23/5
Fibrosis score (F0-F4/F5-F6)	15/39
Child-Pugh score (A/B/C)	54/4/0
BCLC staging (0-A/B/C)	37/14/5
Cirrhosis (Y/N)	45/13
Grading (well/medium/poor and not diff.)	15/28/11
Vascular invasion (Y/N)	14/39

Categorical variables are reported as the number of patients, while continuous variables are reported as median [IQR–IIIQR] or mean ± SD, based on their distribution. AFP, α-fetoprotein; ALD, Alcohol-associated liver disease; ALT, Alanine transaminase; AST, Aspartate aminotransferase; BCLC, Barcelona-clinic liver cancer; BMI, Body mass index; F, Female; HBV, Hepatitis B virus; HCV, Hepatitis C virus; HCC, Hepatocellular carcinoma; HDL, High-density lipoprotein; INR, International normalized ratio; M, Male; MASLD, Metabolic dysfunction-associated steatotic liver disease; N, No; Y, Yes.

The study was approved by the Regional Ethics Committee of Friuli Venezia Giulia (Prot. No. 18854). Written informed consent was obtained from all participants, and procedures complied with the Declaration of Helsinki.

### 2.2 Mouse samples

Heterozygous male C57BL/6J-Tg(ALB1HBV)44Bri/J transgenic (TG) mice and age-matched C57BL/6J wild-type (WT) males were used as experimental and control groups, respectively. TG mice stably express a hepatits B virus (HBV) genome fragment encoding Hepatitis B surface antigen (HBsAg) under the albumin promoter and enhancer (25), and develop progressive liver pathology characterized by chronic inflammation, early injury, pre-neoplastic lesions, early neoplasia, and advanced neoplasia from 3 to 15/18 months of age. The study was designed as a controlled, longitudinal observational analysis across predefined disease stages, with the individual mouse as the experimental unit.

At five predefined time points (3, 6, 9, 12, and 15/18 months of age), 11 TG and 11 WT mice were sacrificed (n = 110). Sample size was defined *a priori* to ensure adequate biological replication, and all animals reaching the predefined time points were included, with no exclusions. Mice were anesthetized by intraperitoneal injection of ketamine (10 mg/kg) and xylazine (5 mg/kg). Under deep anesthesia, blood was collected by intracardiac puncture, followed by cervical dislocation as a secondary physical method to ensure death. All procedures were performed in accordance with Directive 2010/63/EU and the American Veterinary Medical Association (AVMA) Guidelines for the Euthanasia of Animals to minimize suffering.

Liver tissues were collected from at least three liver regions (right and left lobes), kept on ice, and stored at −80 °C. In mice presenting with macroscopically visible nodules (12 and 15/18 months), both tumoral and non-tumoral liver regions were sampled and analyzed separately. Animals were allocated to experimental groups according to genotype, while sample identity was blinded during molecular analyses. Randomization of treatment allocation was not applicable. No additional confounders related to treatment order or cage location were identified. The primary outcome measure was hepatic *Cd274* (*Pd-l1*) mRNA expression across disease stages, while secondary outcomes included comparisons between tumoral, non-tumoral, and WT liver tissues.

All procedures were approved by the Ethical Committee of the University of Trieste and the Italian Ministry of Health (D.M. 57/2012-B) and followed ARRIVE 2.0 guidelines. Animals were clinically healthy at the start of the study and had not undergone any prior experimental procedures. Mice were housed under specific pathogen-free (SPF) conditions in individually ventilated cages with 12-hour light/dark cycles, 20–23 °C temperature, 40–60% relative humidity, and *ad libitum* access to standard chow and water. Animals were routinely monitored, and no unexpected adverse events were observed.

### 2.3 HCC cell lines

Five HCC cell lines were used: JHH6, HuH7, HLE, HLF, and Hep3B. JHH6 (JCRB1030, RRID: CVCL_2788) and HuH7 (JCRB0403, RRID: CVCL_0336) were obtained from the Japan Health Science Research Resources Bank (Tokyo, Japan), while HLE (RRID: CVCL_1281), HLF (RRID: CVCL_2947), and Hep3B (RRID: CVCL_0326) were kindly provided by Dr. Gianelli (National Institute of Gastroenterology S. de Bellis research hospital, Bari, Italy). Cells were cultured according to standard protocols and conditions available in the **Supplementary Material**.

### 2.4 PBMC isolation and activation

Peripheral blood mononuclear cells (PBMCs) were isolated from fresh EDTA-anticoagulated blood from five healthy donors. Written informed consent was obtained from all participants, and the study was conducted in accordance with the Declaration of Helsinki. The protocol was approved by the Regional Ethics Committee of Friuli Venezia Giulia (Prot. No. 18854).

Blood samples were diluted and subjected to density gradient centrifugation for PBMC isolation (see **Supplementary Material**). PBMCs were separated by density gradient centrifugation at 1000 × g for 20 minutes at room temperature without braking. The PBMC layer was collected, washed twice with PBS, and residual erythrocytes were removed using red blood cell lysis solution (see **Supplementary Material**). The average yield was 1.5 ± 0.4 × 10^6^ PBMCs/mL of blood.

Cells were resuspended in RPMI-1640 medium supplemented with 10% FBS, 1% L-glutamine, and 1% penicillin/streptomycin and allowed to rest for 24 hours. For activation, PBMCs were seeded at 1 × 10^6^ cells/mL and stimulated with Dynabeads™ Human T-Activator CD3/CD28 (see **Supplementary Material**) at a 1:1 bead-to-cell ratio. After bead removal, both activated and unstimulated PBMCs were used for co-culture experiments or molecular analyses.

### 2.5 Chemicals and treatments

Detailed information on chemicals and experimental conditions for gene silencing, PD-L1 glycosylation analysis, and PD-L1 protein stability assays is provided in the **Supplementary Materials**.

### 2.6 Gene expression analysis

Frozen human and mouse liver samples were homogenized using stainless steel beads in a Bead ruptor 4 (see **Supplementary Material**) or Potter–Elvehjem grinders. Total RNA was extracted according to standard protocols. RNA concentration and purity were determined spectrophotometrically, and 1 µg of RNA was reverse-transcribed into cDNA. Detailed protocols, reagents, and experimental conditions are described in the **Supplementary Materials**.

qRT-PCR was performed using SsoAdvanced™ universal SYBR^®^ green supermix (1725274, Bio-Rad) on a CFX connect real-time PCR system (Bio-Rad). Relative expression was calculated using the 2^−ΔΔCt^ method. The primer sequences (Metabion International AG) for human (B2M, PD-1, PD-L1, 18S) and mouse (Actb, Cd274, Gapdh) genes are provided in **Table 2**.

**Table 2:** List of the primers used in the study.

Gene	Accession number	Forward primer (5’->3’)	Reverse primer (5’->3’)
B2M	NM_004048.4	GTCTCGCTCCGTGGCCTTA	TGGAGTACGCTGGATAGCCTC
PD-1	NM_005018.3	CCCAAGGCGCAGATCAA	GCACTTCTGCCCTTCTCTCTGT
PD-L1	NM_001314029.2	AAGTCCTGAGTGGTAAGA	CATTAGTTGTTGTGTTGATTC
18S	NR_003286.2	CGTCTGCCCTATCAACTTTCG	GCCTGCTGCCTTCCTTGG
Actb	NM_007393.5	CCTTCTTGGGTATGGAATCCTGTG	CAGCACTGTGTTGGCATAGAGG
Cd274	NM_021893.3	TTGTTCCTCATTGTAGTGT	TATCTTCAACGCCACATT
Gapdh	NM_008084.3	CCAGTATGACTCCACTCACG	CTCGCTCCTGGAAGATGGTG

Human genes: B2M, Beta-2-Microglobulin; PD-1 Programmed Cell Death, PDCD1; PD-L1, Programmed Cell Death Ligand 1, CD274. Mouse genes: Actb, Actin β; Cd274, CD274 antigen, Programmed cell death ligand 1; Gapdh, Glyceraldehyde-3-phosphate dehydrogenase.

Any alternative text (alt text) provided alongside figures in this article has been generated by Frontiers with the support of artificial intelligence and reasonable efforts have been made to ensure accuracy, including review by the authors wherever possible. If you identify any issues, please contact us.

### 2.7 Protein expression

Protein concentrations were measured with a BCA assay. Lysates (50 µg for cell lines and 80 µg for tissue samples) were denatured in Laemmli buffer at 95 °C for 5 min, separated by 12% SDS-PAGE, and transferred to PVDF membranes. Membranes were blocked for 1 h at room temperature (5% BSA in PBS-T for PD-L1; 4% non-fat milk in PBS-T for β-actin), incubated with primary antibodies overnight at 4 °C: PD-L1 (1:1000, see **Supplementary Material**) and β-actin (1:3000, see **Supplementary Material**), followed by HRP-conjugated secondary antibodies (see **Supplementary Material**). Molecular weight determination was performed using the Prestained Protein Ladder (10–180 kDa, see **Supplementary Material**).

Flow cytometry was used to assess surface PD-L1 expression in HCC cells and CD45, CD56, CD8, CD69, CD137, and PD-1 expression in PBMCs, according to protocols and conditions described in the **Supplementary Materials**. Matched IgG isotype controls were used to verify staining specificity.

Immunohistochemistry (IHC) for PD-L1 was performed using two different antibodies according to the standard protocol available in the **Supplementary Material**.

### 2.8 Viability and apoptosis analysis in co-culture

HCC cells were co-cultured with PBMCs at defined effector-to-target (E:T) ratios. Cell viability was assessed using the MTT assay (3-(4,5-dimethylthiazol-2-yl)-2,5-diphenyltetrazolium bromide; see **Supplementary Material**), and absorbance was recorded at 562 nm (see **Supplementary Material**).

Apoptosis was quantified using Annexin V/propidium iodide (PI) staining (see **Supplementary Material**), according to the manufacturer’s instructions. Cells positive for Annexin V and/or PI were considered non-viable.

Real-time, label-free monitoring of cell viability was performed using the Maestro Z system (Axion Biosystems), which continuously measures impedance as a proxy for cell attachment and proliferation. PBMC-induced cytotoxicity was calculated relative to target cells alone, as PBMCs did not alter baseline impedance. For ICI experiments, cell viability was normalized to the IgG isotype control.

### 2.9 DepMap gene expression data and cell line selection

Gene expression data for human liver cancer cell lines were obtained from the Cancer Dependency Map (DepMap) public data release (https://depmap.org/portal/). Log-transformed transcript-per-million expression values [log_2_(TPM + 1)] for protein-coding genes were downloaded from the file OmicsExpressionTPMLogp1HumanProteinCodingGenes.csv. Cell line metadata, including lineage and disease annotations, were retrieved from the Model.csv file.

Liver-derived cancer cell lines were selected based on OncotreeLineage = “Liver”. Both HCC cell lines and hepatoblastoma cell lines (e.g., HepG2, HUH6) were included to enable comparative analyses. Gene expression matrices were subset to retain only the selected liver cancer cell lines and genes involved in DNA repair pathways. Expression values were z-score normalized across cell lines to facilitate comparison of relative gene expression levels between models.

All analyses were performed using R (RRID: SCR_001905, version ≥ 4.2.0) within the RStudio environment (RRID: SCR_000432), using the following packages: base R, dplyr, stringr, and pheatmap. Heatmaps were generated using the pheatmap package. Hierarchical clustering was applied to both genes (columns) and cell lines (rows) using Euclidean distance and complete linkage. Scaled expression values were visualized using a diverging color palette ranging from low (navy) to high (firebrick red) expression.

The heatmap was used to assess inter–cell line heterogeneity in DNA repair gene expression and to identify potential clustering patterns among HCC–derived models.

### 2.10 Database and statistical analysis

Gene and protein expression data were obtained from Gene expression profiling interactive analysis (GEPIA; RRID: SCR_018294, http://gepia.cancer-pku.cn/), DepMap (RRID: SCR_017655, https://depmap.org/portal/), and Cancer cell line encyclopedia (CCLE) proteomics.

Statistical analyses were performed using GraphPad Prism version 9.2.0 (RRID: SCR_002798, GraphPad Software). Data distribution was assessed using the Shapiro–Wilk test for normality. For normally distributed data, parametric tests were applied, including one-sample t-tests or two-tailed unpaired t-tests, as appropriate. For non-normally distributed data, non-parametric tests were used, including the Wilcoxon matched-pairs signed-rank test, Mann–Whitney U test, or Spearman’s rank correlation analysis. GEPIA applies an ANOVA-based method for comparison. Categorical variables were analyzed using the chi-square test.

For real-time impedance–based assays, outliers among technical replicates were identified and excluded using Grubbs’ test (α = 0.1). Data are presented as mean ± standard deviation (SD) for parametric analyses or as median [interquartile range (IQR)] for non-parametric analyses. Statistical significance was defined as p < 0.05.

## 3 Results

### 3.1 The complex landscape of PD-L1 expression in HCC

**Figure 1:**
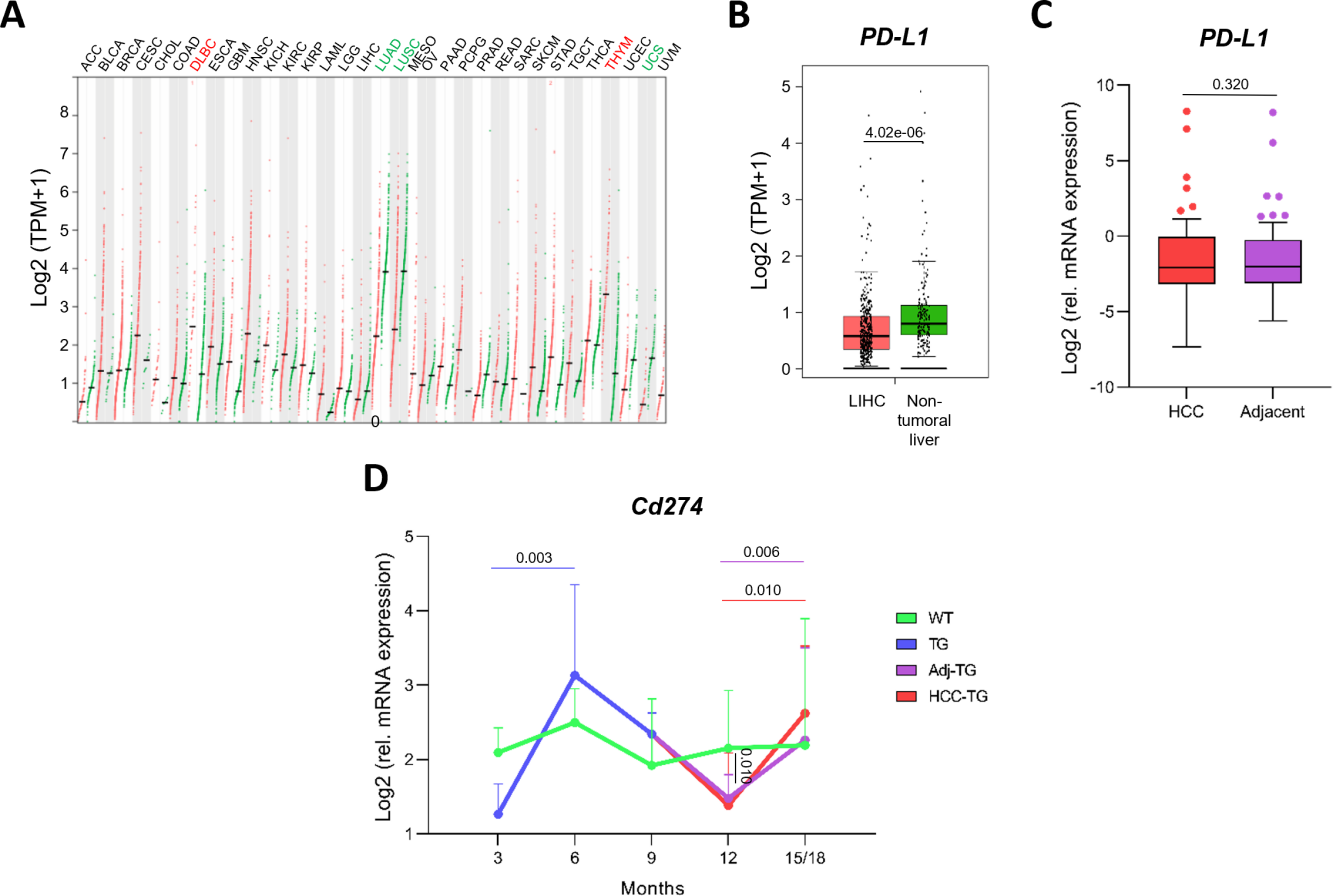
*PD-L1* (*CD274*) mRNA expression in HCC. **(A)***PD-L1* mRNA expression in tumor (T; red) and non-tumoral (N; green) tissues across cancer types, as retrieved from the GEPIA database. **(B)***PD-L1* mRNA expression in liver hepatocellular carcinoma (LIHC; n = 369) compared with normal liver tissues (n = 160). Statistical significance was evaluated using an ANOVA test. **(C)** Relative *PD-L1* mRNA expression in HCC tissues and paired adjacent non-tumoral liver tissues (n = 54). Statistical significance was evaluated using the Wilcoxon matched-pairs signed-rank test. **(D)** Age-associated *Cd274* mRNA median ± interquartile range expression levels in transgenic (TG) and wild-type (WT) mice at 3, 6, 9, 12, and 15–18 months of age (n = 11 per group). Statistical significance was evaluated using the Mann-Whitney test. < < < ACC, Adrenocortical Carcinoma (T [n = 77] *vs.* D [n = 128]); BLCA, Bladder Urothelial Carcinoma (404 *vs.* 28); BRCA, Breast Invasive Carcinoma (1085 *vs.* 291); CESC, Cervical Squamous Cell Carcinoma And Endocervical Adenocarcinoma (306 *vs.* 13); CHOL, Cholangiocarcinoma (36 *vs.* 9); COAD, Colon Adenocarcinoma (275 *vs.* 349); DLBC, Lymphoid Neoplasm Diffuse Large B-Cell Lymphoma (46 *vs.* 337); ESCA, Esophageal Carcinoma (182 *vs.* 286); GBM, Glioblastoma Multiforme (163 *vs.* 207); HNSC, Head And Neck Squamous Cell Carcinoma (519 *vs.* 44); KICH, Kidney Chromophobe (66 *vs.* 53); KIRC, Kidney Renal Clear Cell Carcinoma (523 *vs.* 100); KIRP, Kidney Renal Papillary Cell Carcinoma (286 *vs.* 60); LAML, Acute Myeloid Leukemia (173 *vs.* 70); LGG, Brain Lower Grade Glioma (518 *vs.* 207); LIHC, Liver Hepatocellular Carcinoma (369 *vs.* 160); LUAD, Lung Adenocarcinoma (483 *vs.* 347); LUSC, Lung Squamous Cell Carcinoma (486 *vs.* 338); MESO, Mesothelioma (T: n = 87); OV, Ovarian Serous Cystadenocarcinoma (T [n = 426] *vs.* D [n = 88]); PAAD, Pancreatic Adenocarcinoma (179 *vs.* 171); PCPG, Pheochromocytoma And Paraganglioma (182 *vs.* 3); PRAD, Prostate Adenocarcinoma (492 *vs.* 152); READ, Rectum Adenocarcinoma (92 *vs.* 318); SARC, Sarcoma (262 *vs.* 2); SKCM, Skin Cutaneous Melanoma (461 *vs.* 558); STAD, Stomach Adenocarcinoma (406 *vs.* 211); TGCT, Testicular Germ Cell Tumors (137 *vs.* 165); THCA, Thyroid Carcinoma (512 *vs.* 337); THYM, Thymoma (118 *vs.* 339); UCEC, Uterine Corpus Endometrial Carcinoma (174 *vs.* 91); UCS, Uterine Carcinosarcoma (57 *vs.* 78); UVM, Uveal Melanoma (T: n = 79).

Although PD-L1 expression has been reported in HCC for several years, recent analyses reveal a highly heterogeneous landscape, with substantial variability across patient cohorts, detection methods, and underlying disease etiologies (26–28). Analysis of GEPIA datasets indicates that approximately two-thirds of tumor types display an overall increase in *PD-L1* (*CD274*) mRNA expression, although in most cases the difference between tumor and non-tumoral tissues is negligible (**Figure 1A**). Among the remaining cancer types, *PD-L1* is significantly decreased in lung adenocarcinoma (LUAD), lung squamous cell carcinoma (LUSC), and uterine carcinosarcoma (UCS), while increased expression is seen in thymoma (THYM) and diffuse large B-cell lymphoma (DLBC; **Figure 1A**).

In HCC, it appears only marginally reduced compared to non-tumoral liver tissue, showing a modest 1.36-fold decrease (0.58 *vs.* 0.79, p = 0.42e-06, **Figure 1B**). Importantly, differences in *PD-L1* expression across tumor types may reflect not only intrinsic biological features of each tissue but also the nature of the reference samples used for comparison, which range from healthy tissue to paired tissue affected by chronic underlying disease, as is frequently the case in HCC.

Consistent with GEPIA data, analysis of our cohort showed no significant difference in *PD-L1* mRNA expression between HCC and paired adjacent non-tumoral tissues (0.23 [0.11-0.99] *vs*. 0.25 [0.12-0.85], p = 0.370; **Figure 1C**), nor any significant association with OS or disease-free survival (DFS) (**Supplementary Figures 1A–D**). Nevertheless, lower PD-L1 expression in tumor tissue was associated with a trend toward poorer outcomes, including a 2.08-fold increased risk of reduced OS (p = 0.099; **Supplementary Figure 1A**) and a 2.07-fold increased risk of early recurrence (p = 0.066; **Supplementary Figure 1B**).

Analysis of a TG mouse model of chronic HBV-driven hepatocarcinogenesis further supported these observations. At the time of tumor development (12 months), early neoplastic lesions display *Cd274 (Pd-l1*) mRNA levels (1.38 [1.01-2.09]) that are almost identical to those of their paired non-tumoral counterparts (1.48 [1.04-1.80]), p = 0.700; **Figure 1D**). Notably, both tumor and adjacent tissues displayed significantly lower Cd274 expression than healthy WT mice (2.15 [1.62-2.93]). Interestingly, *Cd274* expression rose during early hepatic injury (6 months *vs.* 3 months: 3.13 [2.01-4.35] *vs.* 1.27 [0.53-1.67], p = 0.003; **Figure 1D**), possibly reflecting disease-dependent inflammatory activation during the disease progression. This pattern parallels the PD-L1 upregulation described in HBV and HCV infections in humans (29, 30).

### 3.2 PD-L1 is upregulated in HCC compared to paired non-tumoral tissue

**Figure 2:**
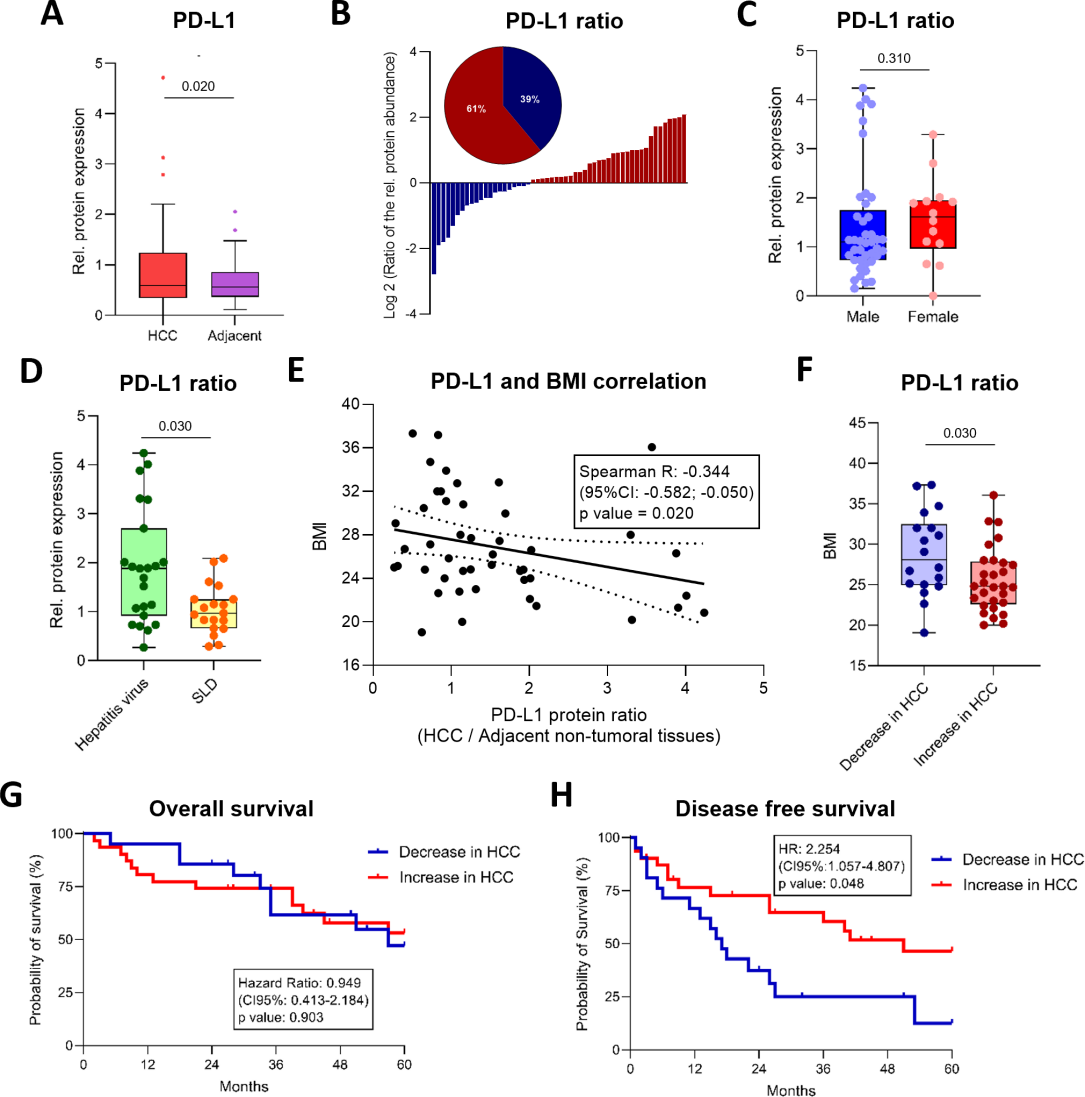
PD-L1 protein expression in HCC. **(A)** Relative PD-L1 protein expression in HCC tissues and paired adjacent non-tumoral liver tissues (n = 55). Statistical significance was evaluated using the Wilcoxon matched-pairs signed rank test. **(B)** Tumor-to-adjacent tissue ratios of PD-L1 protein expression across the cohort (n = 55). **(C, D)** Tumor-to-adjacent tissue ratios of PD-L1 expression stratified by gender [**(C)** females, n = 14 *vs*. males, n = 41] and disease etiology [**(D)** viral, n = 23 *vs*. steatotic liver disease (SLD), n = 20]. Statistical significance was evaluated using the Mann-Whitney test. **(E)** Correlation between tumor-to-adjacent tissue PD-L1 expression ratios and body mass index (BMI; n = 46). **(F)** BMI distribution in patients with tumors exhibiting increased *vs.* decreased PD-L1 expression. Statistical significance was evaluated using the Mann-Whitney test. **(G, H)** Overall survival [OS; **(G)**] and disease-free survival [DFS; **(H)**] curves stratified by tumors with increased *vs.* decreased PD-L1 expression.

To better define PD-L1 regulation at the protein level, we systematically quantified PD-L1 expression in human HCC samples and matched adjacent non-tumoral liver tissues. Total PD-L1 protein levels were modest but significantly increased in tumors compared with paired adjacent tissue (0.591 [0.341-1.244] *vs.* 0.562 [0.366-0.861]; p = 0.020; **Figure 2A**), supporting tumor-specific upregulation rather than inter-patient baseline variability. Paired analysis further showed a significant increase in 61% of cases (p = 0.020; **Figure 2B**).

Tumor-to-adjacent PD-L1 ratios did not differ significantly by gender, although median values were higher in females than in males (1.61 [0.97-1.95] *vs.* 1.10 [0.73-1.75]; p = 0.310; **Figure 2C**), with PD-L1 upregulation observed in 79% of women compared with 54% of men. Etiology influenced PD-L1 modulation, as viral hepatitis–related HCC showed a higher frequency and magnitude of PD-L1 upregulation than steatotic liver disease (SLD)-associated HCC (73% *vs.* 47%; ratio 1.88 [0.92-2.70] *vs*. 0.97 [0.66-1.25]; p = 0.030; **Figure 2D**), consistent with enhanced immune-selective pressure in virally driven tumors.

PD-L1 variation was also associated with metabolic status. The tumor–adjacent difference inversely correlated with body mass index (BMI) (Spearman r = –0.344 (95% CI: –0.582; –0.050); p = 0.020; **Figure 2E**), and patients with decreased tumoral PD-L1 had significantly higher BMI than those with PD-L1 upregulation (28.1 [25.0-32.5] *vs.* 24.8 [22.6-27.9]; p = 0.030; **Figure 2F**). Tumor-to-adjacent PD-L1 ratios were not associated with OS (**Figure 2G**), while higher PD-L1 levels in adjacent non-tumoral tissue were associated with a 2.25-fold increased risk of recurrence (p = 0.048; **Figure 2H**), suggesting that sustained PD-L1 expression in the residual liver may contribute to a microenvironment permissive to tumor relapse.

### 3.3 PD-L1 is heavily N-glycosylated in HCC with 33–37 kDa species representing newly synthesized and proteasome-regulated PD-L1

**Figure 3:**
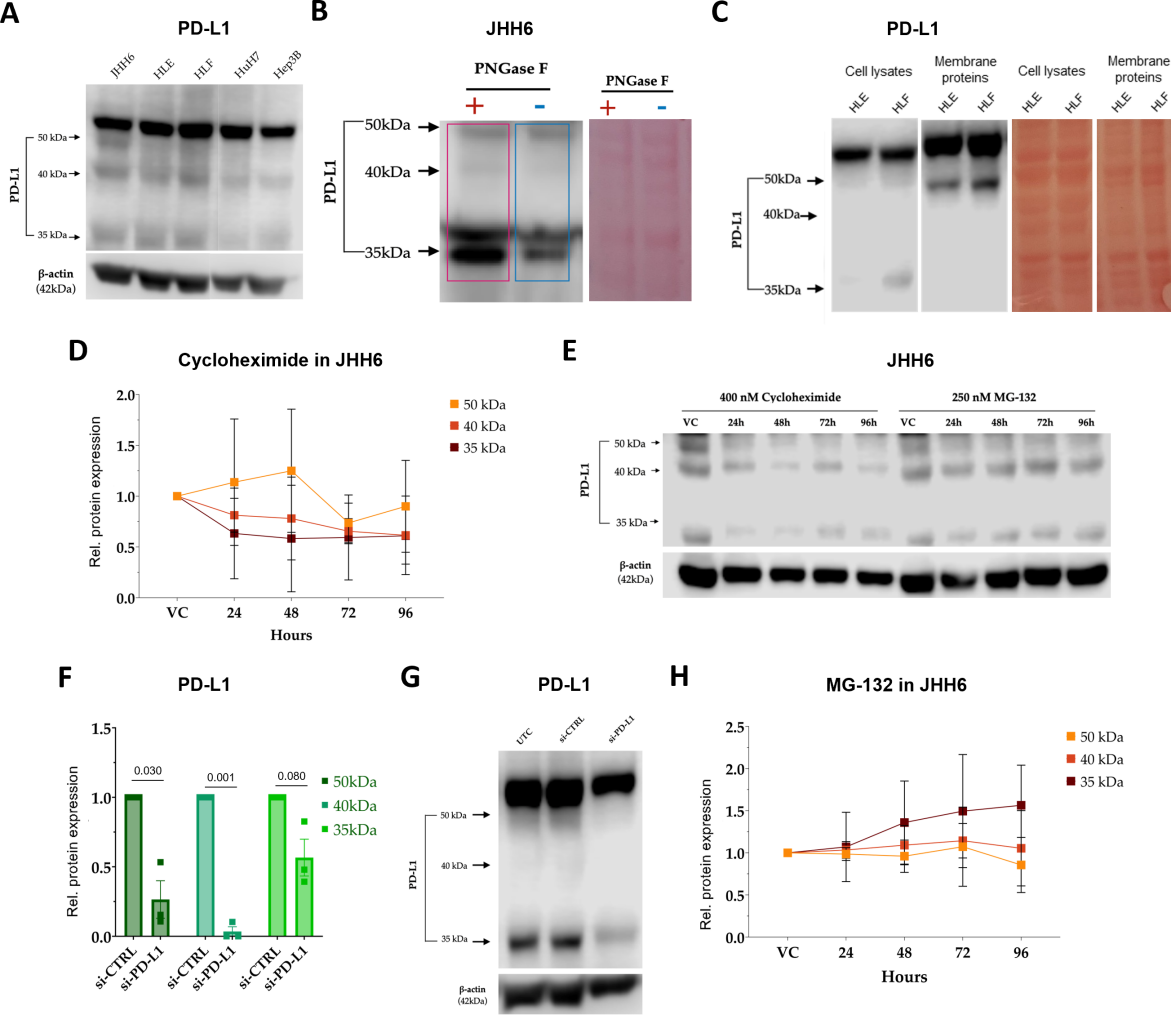
PD-L1 displays multiple biochemical species in HCC cells with distinct glycosylation, subcellular localization, and proteasome-dependent regulation. **(A)** Representative immunoblots showing PD-L1 expression in multiple HCC cell lines, revealing distinct bands at ~33–37 kDa, ~39–45 kDa, and ~50 kDa. **(B)** PNGase F treatment in JHH6 cells demonstrating PD-L1 de-glycosylation (red box). Ponceau Red staining is shown as a loading control. **(C)** Comparison of PD-L1 band distribution in crude membrane fractions *vs.* whole-cell lysates from HCC cell lines; the ~50 kDa band corresponds to the membrane-associated PD-L1 species. Ponceau Red staining is shown as a loading control. **(D)** Relative PD-L1 expression following cycloheximide (CHX) chase (24–96 h) in JHH6 cells (n = 3). VC, vehicle control. **(E)** Representative immunoblots resolving the ~33–37 kDa, ~39–45 kDa, and ~50 kDa PD-L1 species in JHH6 cells following CHX chase or MG-132–mediated proteasome inhibition. **(F, G)** Quantification of ~33–37 kDa, ~39–45 kDa, and ~50 kDa PD-L1 protein levels **(F)** and representative immunoblot **(G)** following 72-hour siRNA-mediated PD-L1 silencing in JHH6 cells (n = 3). Statistical significance was evaluated using a one-sample t-test. UTC, untreated control. **(H)** Relative PD-L1 expression following MG-132–mediated proteasome inhibition (24–96 h) in JHH6 cells (n = 3). VC, vehicle control.

Previous studies in non-hepatic tumors have demonstrated that PD-L1 undergoes extensive post-translational modification, predominantly N-linked glycosylation, which regulates its stability, trafficking, and immune checkpoint activity (9, 31). Immunoblot analysis of HCC-derived cell lines revealed multiple PD-L1 species migrating at ~33–37 kDa, ~39–45 kDa, and ~50 kDa (**Figure 3A**). These bands were consistently detected across cell lines and included the membrane-associated form responsible for immune checkpoint function. Enzymatic de-glycosylation and molecular weight comparisons confirmed that the ~50 kDa species corresponds to the fully glycosylated (**Figure 3B**, **Supplementary Figure 2A**), membrane-localized form of PD-L1 (**Figure 3C**; **Supplementary Figure 2B**), whereas the ~39–45 kDa and ~33–37 kDa bands represent progressively less glycosylated, immature precursor forms (**Figure 3B**, **Supplementary Figure 2A**).

Cycloheximide chase experiments in JHH6 cells demonstrated a clear maturation hierarchy: the ~33–37 kDa and ~39–45 kDa species declined rapidly, whereas the fully mature ~50 kDa form exhibited delayed turnover, with reduction becoming evident only after 72 h (**Figures 3D, E**). Consistently, PD-L1 silencing reduced all molecular species at 72 h, predominantly affecting the more glycosylated ~39–45 kDa and ~50 kDa forms (**Figures 3F, G**, **Supplementary Figures 2C, D**) as well as membrane-localized PD-L1 (**Supplementary Figure 2E**), suggesting that the reduction of the glycosylated species was not offset by maturation of the limited un-glycosylated, newly synthetized species. Proteasome inhibition resulted in approximately 50% accumulation of the ~33–37 kDa species after 72 h, while mature forms remained largely unchanged up to 96 h (**Figures 3E, 3H**), indicating that newly synthesized PD-L1 is preferentially targeted for degradation.

Together, these data identify a dynamic, multi-step PD-L1 maturation process in HCC cells, with immature PD-L1 tightly controlled by proteasomal turnover. This phenomenon, largely unexplored in liver cancer, could be relevant for therapeutic targeting of PD-L1 stability and function.

### 3.4 HCC tissues display multiple PD-L1 molecular species corresponding to distinct glycosylation states

Despite extensive investigation, PD-L1 expression in HCC remains incompletely characterized, partly due to substantial inter-tumor heterogeneity and the co-existence of multiple molecular forms of PD-L1. Such complexity may obscure biologically relevant patterns when only total protein levels are considered.

**Figure 4:**
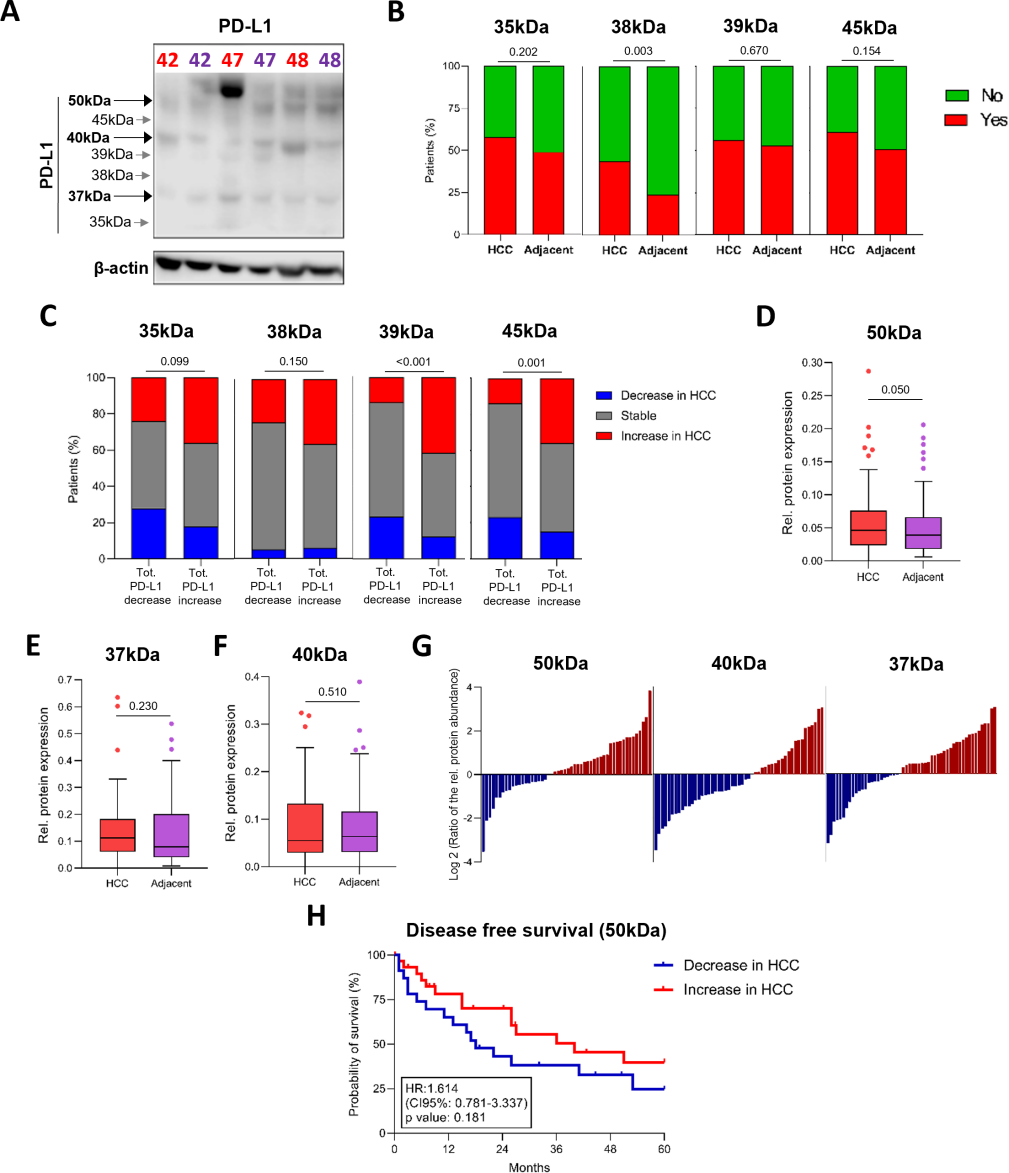
PD-L1 expression displays multiple biochemical species in HCC. **(A)** Representative immunoblot showing PD-L1 protein expression in HCC (red) and paired adjacent non-tumoral liver tissues (purple). **(B)** Presence (red) or absence (green) of PD-L1 bands at 35, 38, 39, and 45 kDa across the cohort (n = 55). Statistical significance was evaluated using the Chi-square test. **(C)** Percentage of patients exhibiting increased (blue), stable (grey), or decreased (green) intensity of individual PD-L1 bands (35, 38, 39, and 45 kDa) in HCC tissues, stratified by cases with overall increased *vs.* decreased total PD-L1 expression. Statistical significance was evaluated using the Chi-square test. **(D–F)** Relative expression levels of individual PD-L1 bands (n = 55): 50 kDa **(D)**, 37 kDa **(E)**, and 40 kDa **(F)**. Statistical significance was evaluated using the Wilcoxon matched-pairs signed-rank test. **(G)** Tumor-to-adjacent tissue ratios of the indicated PD-L1 bands (50, 40, and 37 kDa). **(H)** DFS stratified by tumors with increased *vs.* decreased 50 kDa PD-L1 expression.

In human HCC samples, PD-L1 heterogeneity appears even more pronounced than in cell lines. In addition to the predominant bands at ~50 kDa, ~40 kDa, and ~37 kDa, several additional species were detected at ~45, ~39, ~38, and ~35 kDa (**Figure 4A**). These lower-abundance forms were more frequently observed in tumor tissue than in matched adjacent liver (~38 kDa: 44% *vs.* 24%, p = 0.003; **Figure 4B**). The ~45 kDa and ~39 kDa species were significantly enriched in tumors exhibiting overall PD-L1 upregulation (45 kDa: 36% *vs.* 14%, p = 0.001; 39 kDa: 42% *vs.* 14%, p < 0.001; **Figure 4C**).

Within tumor nodules, the fully mature ~50 kDa band was increased in 62% of cases (p = 0.050; **Figures 4D, G**), while the ~37 kDa precursor was elevated in 57% (**Figures 4E, G**). Conversely, the ~40 kDa species was more prevalent in adjacent non-tumoral tissue (59%; **Figures 4F, G**). Although not statistically significant, the ~50 kDa band showed the strongest association with DFS, and higher PD-L1 levels in adjacent tissue were associated with an increased risk of recurrence (HR 1.61, p = 0.181; **Figure 4H**).

The ~50 kDa PD-L1 species positively correlated with serum albumin levels (Spearman r = 0.288; 95% CI 0.019–0.518; p = 0.030; **Supplementary Figure 3A**) and was significantly enriched in virally associated HCC compared with SLD-related tumors (p = 0.005), indicating an association between highly glycosylated PD-L1 forms and viral etiology. The ~40 kDa PD-L1 band correlated with total bilirubin levels (Spearman r = 0.400; 95% CI 0.140–0.608; p = 0.003; **Supplementary Figure 3B**), whereas the ~37 kDa species correlated with glutathione S-transferase omega 1 (GSTO1) expression (Spearman r = 0.423; 95% CI 0.088–0.671; p = 0.010; **Supplementary Figure 3C**) and telomere length in adjacent non-tumoral tissues (Spearman r = 0.493; 95% CI 0.089–0.758; p = 0.020; **Supplementary Figure 3D**).

Collectively, these associations support the existence of tumor- and patient-specific PD-L1 glycoprofiles, which may reflect differences in PD-L1 trafficking and maturation and potentially influence its functional state within the hepatic tumor microenvironment.

### 3.5 Differential PD-L1 glycosylation affects immunohistochemical detection

Despite increased membrane-associated PD-L1 in a substantial fraction of HCCs, its detection by IHC was highly antibody-dependent. Comparison of HCC samples classified as PD-L1–low or PD-L1–high by western blot using clone 2B11D11 (**Supplementary Figure 4A**) revealed marked discrepancies in IHC staining depending on the antibody employed. Antibodies recognizing unglycosylated epitopes detected high PD-L1 expression, with clone 2B11D11, targeting the extracellular domain, revealing elevated PD-L1 levels in both tumor and adjacent tissues, consistent with recognition of multiple PD-L1 molecular species (**Supplementary Figure 4B**).

However, these approaches are suboptimal for clinical decision-making, as total PD-L1 abundance did not reliably reflect membrane-localized PD-L1. In HuH7 and Hep3B cell lines, multiple PD-L1 species were detected by western blot (**Figure 3A**), whereas only minimal amounts of the fully glycosylated ~50 kDa PD-L1 species, corresponding to membrane-associated PD-L1, were observed (**Figure 3C**, **Supplementary Figure 2B**).

Conversely, antibodies whose epitopes are masked by specific glycosylation states underestimate PD-L1 expression. The SP263 clone, widely used for immunotherapy stratification in non-small cell lung cancer and targeting the cytoplasmic domain, consistently yielded low PD-L1 scores (0–1%) in both tumor and adjacent tissues, with positivity largely confined to macrophage populations (**Supplementary Figure 4C**).

Together with previous reports (32), these findings demonstrate that glycosylation-dependent epitope masking substantially affects PD-L1 immunodetection in HCC and underscore the need for glyco-aware PD-L1 assessment strategies.

### 3.6 Reconstructing tumor-like PD-L1 complexity in HCC cell lines

To evaluate whether hepatoma cell lines recapitulate the molecular heterogeneity of PD-L1 observed in human HCC, we first analyzed transcriptomic and proteomic datasets across major liver cancer models. Establishing whether *in vitro* systems mirror the complexity of PD-L1 maturation *in vivo* is crucial for dissecting its regulatory dynamics and for developing reliable cellular platforms to study PD-L1 biology. This knowledge is also essential for designing robust assays to evaluate responses to ICIs, whose efficacy may depend on the specific PD-L1 isoforms and subcellular trafficking states present in tumor cells. HCC-derived cell lines have been stratified into the well-characterized Hoshida S1 and S2 subclasses (S3 was not observed) (33), originally defined by transcriptomic profiling of primary tumors and reflecting distinct biological programs and clinical behaviors (13).

**Figure 5:**
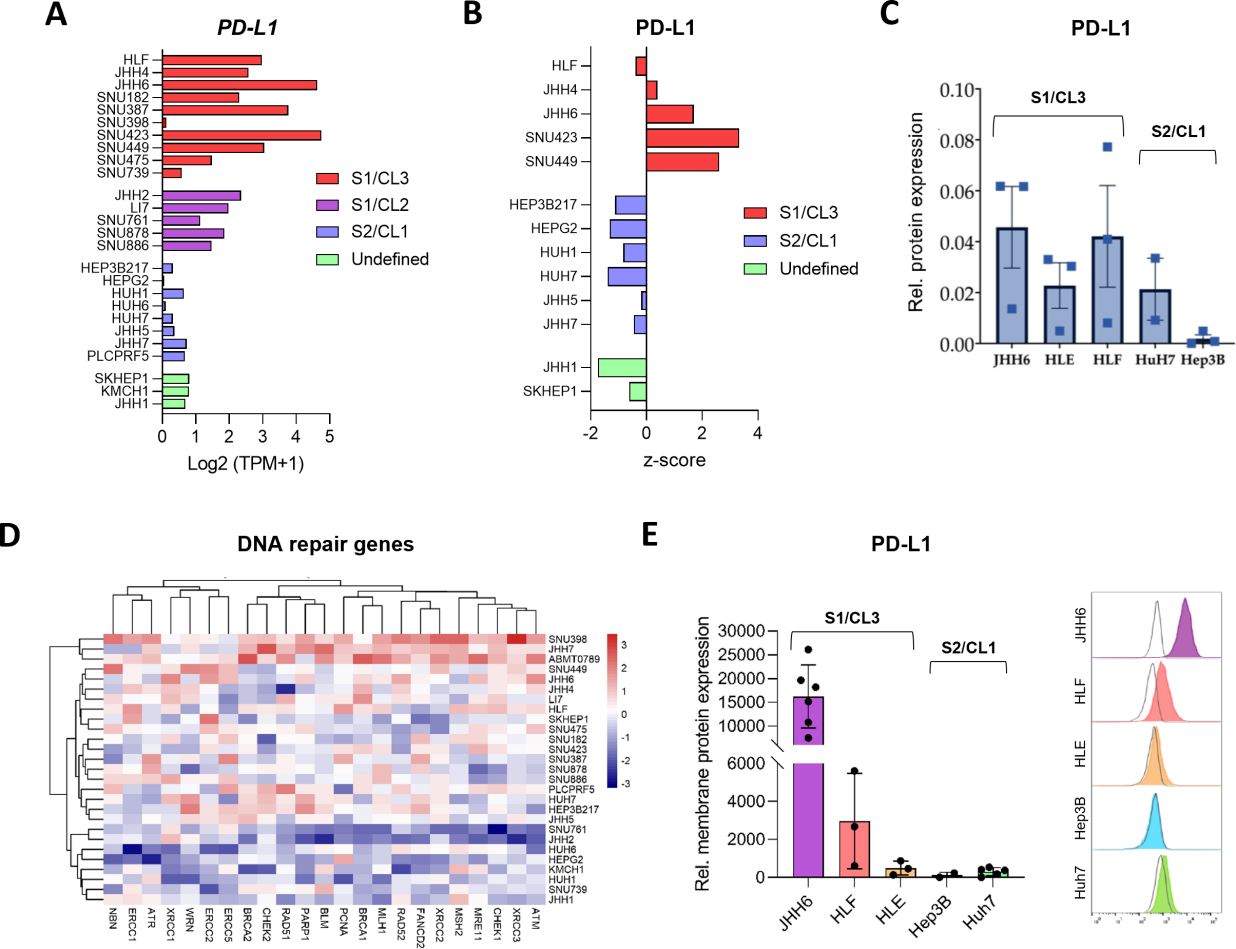
PD-L1 expression across HCC cell lines. **(A)***PD-L1* (*CD274*) mRNA expression in HCC cell lines, derived from the DepMap 25Q3 dataset. **(B)** PD-L1 protein expression in HCC cell lines, as reported in the CCLE proteomics dataset (34). **(C)** Relative quantification of total PD-L1 protein abundance in JHH6, HLE, HLF, HuH7, and Hep3B cell lines (n = 3). **(D)** Heatmap showing expression of DNA repair–related genes across HCC cell lines. **(E)** Relative membrane PD-L1 expression measured by flow cytometry (n ≥ 2), normalized to isotype control (grey).

DepMap analysis revealed that *PD-L1* mRNA expression was highest in cell lines of the Hoshida S1 and Caruso CL3 subclasses, with SNU423 and JHH6 exhibiting the strongest levels, whereas HepG2 and HuH6 showed the lowest expression (**Figure 5A**), despite the absence of copy number variations (**Supplementary Figure 5A**). Gene dependency scores from RNAi and CRISPR screens were near zero across all lines, indicating PD-L1 is dispensable for proliferation and survival *in vitro* (**Supplementary Figure 5B**). Proteomic profiling confirmed elevated PD-L1 protein abundance in S1/CL3 lines (SNU423, SNU449, JHH6) relative to S2/CL1 models (HuH7, Hep3B, HepG2; **Figure 5B**).

Western blot analysis revealed multiple PD-L1 species (~33–37 kDa, ~39–45 kDa, ~50 kDa) with distinct patterns across five representative lines (**Figure 3A**). Total PD-L1 expression was highest in JHH6 and HLF, both belonging to the S1/CL3 subclass, intermediate in HLE and HuH7, and lowest in Hep3B (S2/CL1; **Figures 3A**, **5C**). Beyond its canonical immune-checkpoint role, PD-L1 has been implicated in tumor-intrinsic processes, including regulation of DNA repair (31, 35). Consistently, DNA repair–related gene expression was elevated in S1 subclass lines (**Figure 5D**), suggesting that high PD-L1 levels in this molecular subtype may support tumor-intrinsic resistance mechanisms.

Flow cytometry of non-permeabilized cells using two independent antibodies showed maximal surface PD-L1 in JHH6, low in HuH7, and minimal in Hep3B (**Figure 5E**, **Supplementary Figure 5C**). Consistent with this, HuH7 and Hep3B predominantly retained PD-L1 intracellularly or in the nucleus, supporting potential non-canonical roles in RNA regulation (36, 37) or cell cycle control (38), whereas intracellular PD-L1 in S2 cells did not correlate with DNA repair (**Figure 5D**).

Collectively, these results indicate that S1/CL3 hepatoma lines faithfully model the complex PD-L1 maturation and functional heterogeneity observed in human HCC, providing a robust system for mechanistic studies and preclinical evaluation of ICIs.

### 3.7 Subtype-dependent PD-L1 turnover and maturation dynamics in HCC

**Figure 6:**
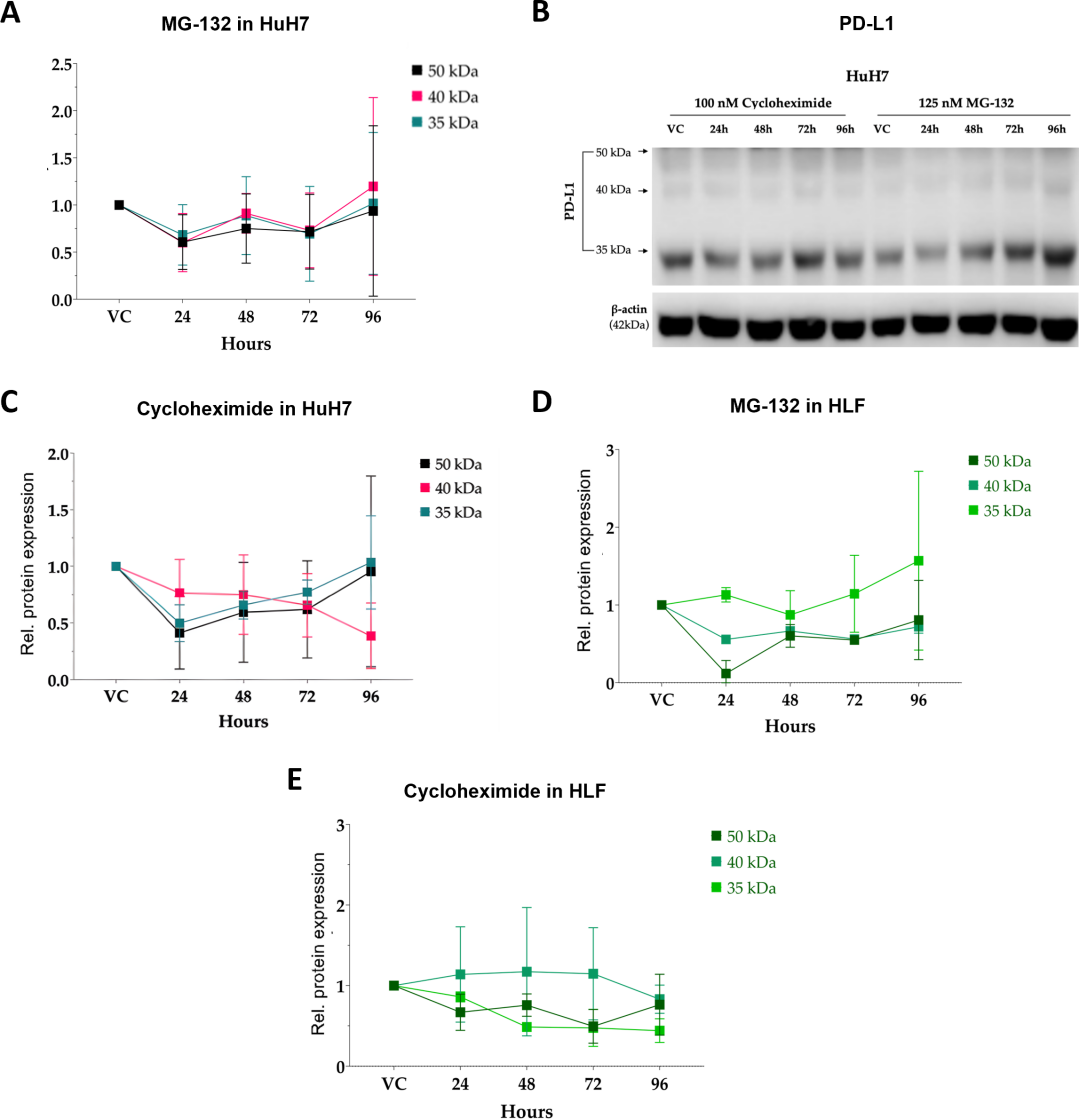
Distinct PD-L1 turnover and proteasome dependence in HuH7 and HLF cells. **(A)** Relative PD-L1 protein expression following MG-132–mediated proteasome inhibition (24–96 h) in HuH7 cells (n = 3). VC, vehicle control. **(B)** Representative immunoblots resolving the ~33–37 kDa, ~39–45 kDa, and ~50 kDa PD-L1 species in HuH7 cells. **(C)** Relative PD-L1 protein expression following CHX chase (24–96 h) in HuH7 cells (n = 3). VC, vehicle control. **(D, E)** Relative PD-L1 protein expression following MG-132–mediated proteasome inhibition **(D)** or CHX chase **(E)** (24–96 h) in HLF cells (n = 3). VC, vehicle control.

Although PD-L1 glycosylation heterogeneity has been reported, the temporal relationship between immature and mature PD-L1 species in HCC cells remains poorly defined. While individual cell lines displayed distinct regulatory patterns (**Supplementary Figures 6A, B**), a key feature distinguishing S1/CL3 from S2/CL1 cells was the coordinated behavior of PD-L1 species observed in the latter group (**Figures 6A–C**, **Supplementary Figures 6C, D**). In S2/CL1 cells, proteasomal inhibition induced a slow and homogeneous accumulation of all PD-L1 forms, with a progressive increase reaching approximately 50% at 96 h, most prominently in Hep3B cells (**Supplementary Figure 6C**). In HuH7 cells, PD-L1 levels remained largely stable over time, suggesting that upon blockade of protein degradation, these cells compensate by limiting *de novo* protein synthesis to maintain homeostasis (**Figures 6A, B**).

Conversely, inhibition of protein synthesis induces a rapid reduction in PD-L1 abundance within 24h, particularly affecting the ~50 kDa and ~33–37 kDa species, indicating that S2/CL1 cells predominantly rely on continuous *de novo* synthesis rather than post-translational stabilization to maintain PD-L1 levels. Under protein synthesis blockade, PD-L1 levels partially recovered by 96 h, possibly reflecting a stress-induced attenuation of degradation pathways aimed at restoring equilibrium (**Figures 6B, C**, **Supplementary Figure 6D**). Notably, the 40–45 kDa band displays a persistent decrease, exceeding 50% in HuH7 cells at 96 h, further supporting limited post-translational buffering of mature PD-L1 in these immune-cold models (**Figures 6B, C**).

In contrast, S1/CL3 cells, which naturally express higher PD-L1, exhibited a distinct regulatory pattern. Upon proteasomal inhibition, the newly synthesized ~33–37kDa PD-L1 species accumulate progressively, increasing by more than 50% between 72 and 96 h across all S1/CL3 cell lines, whereas the mature forms remain largely unchanged (**Figures 3E, H**, **6D**, **Supplementary Figure 6E**). This suggests that S1/CL3 cells have robust post-translational mechanisms capable of stabilizing membrane-associated PD-L1 even when protein degradation is impaired, thereby allowing selective expansion of the immature PD-L1 pool. On the contrary, inhibition of protein synthesis led to a marked reduction (~50% at 72 h) in mature membrane PD-L1 species, confirming their reliance on continuous synthesis to sustain high surface expression (**Figures 3D, E**, **6E**, **Supplementary Figure 6F**).

Collectively, these results identify two distinct PD-L1 regulatory programs in HCC. S2/CL1 cells primarily depend on ongoing *de novo* PD-L1 synthesis with limited post-translational buffering, whereas S1/CL3 cells preserve membrane-bound PD-L1 through enhanced post-translational stabilization, enabling rapid accumulation of newly synthesized intermediates under proteotoxic stress. These subtype-specific dynamics are likely to influence baseline immune evasion and may critically shape responsiveness to PD-L1–targeting therapies.

### 3.8 PD-L1–high S1 cells remain susceptible to CD8^+^-mediated cytotoxicity following checkpoint blockade

A critical challenge in cancer immunotherapy is identifying patients most likely to benefit from immune checkpoint blockade. Although PD-L1 is classically associated with immune evasion, tumors with high PD-L1 expression paradoxically exhibit greater clinical responsiveness to PD-1/PD-L1 inhibitors, with marked interpatient variability. To investigate the mechanistic basis of this paradox in HCC, we established co-culture systems using S1-type (JHH6) and S2-type (HuH7) HCC cells with primary CD8^+^ T cells.

**Figure 7:**
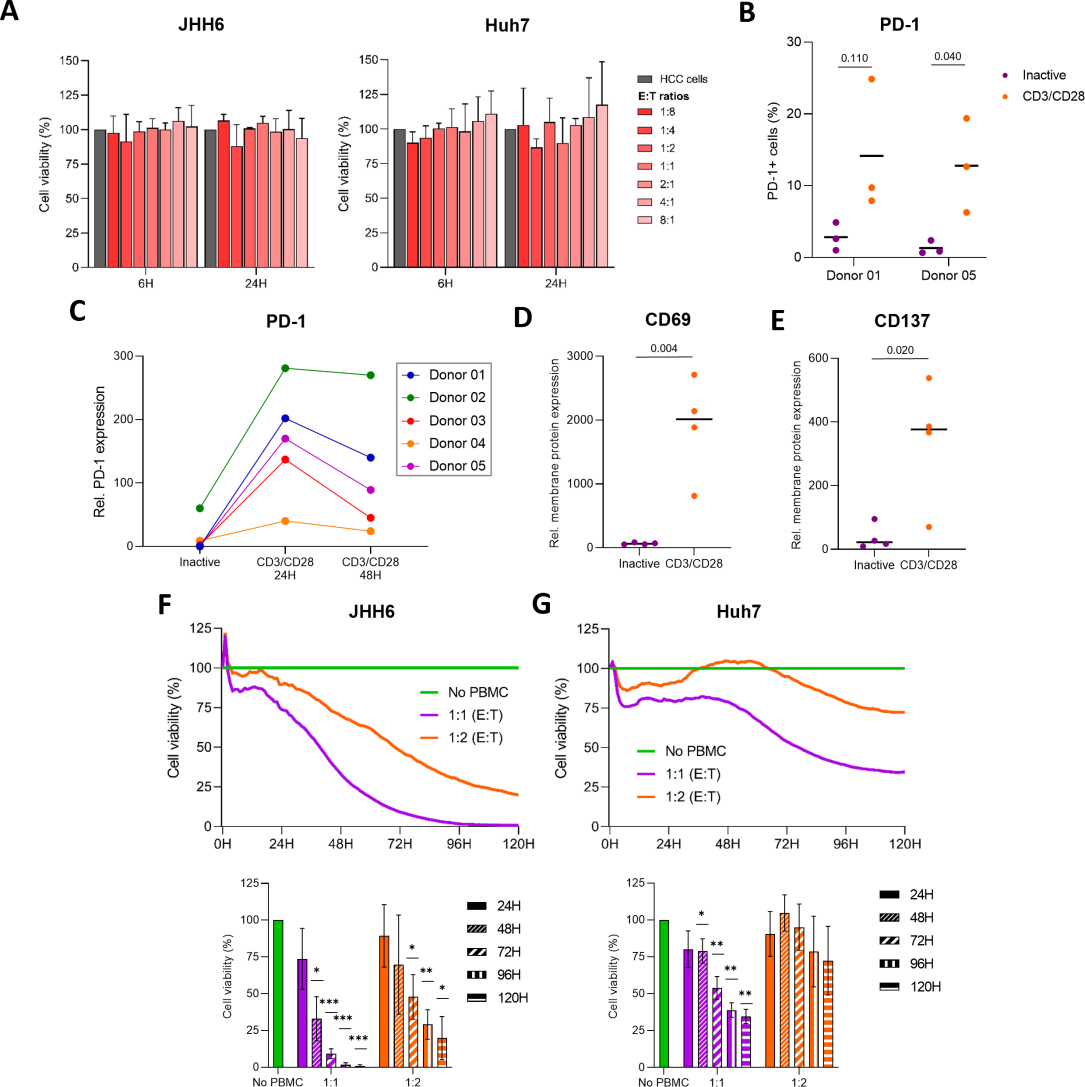
Effects of immune cell co-culture on HCC cell viability. **(A)** Effects of co-culture with immune effector cells at increasing effector-to-target (E:T) ratios on the viability of JHH6 and HuH7 cells (n = 3). **(B, C)** PD-1 membrane expression on non-activated *vs.* activated CD8^+^ T cells, assessed by flow cytometry. Statistical significance was evaluated using an unpaired t-test. **(D, E)** Relative membrane expression of the activation markers CD69 **(D)** and CD137 **(E)** on non-activated and CD3/CD28-activated CD8^+^ T cells, as determined by flow cytometry. Statistical significance was evaluated using an unpaired t-test. **(F, G)** Impedance-based assessment of JHH6 **(F)** and HuH7 **(G)** cell viability during co-culture with activated PBMCs at the indicated E:T ratios. Statistical significance was evaluated using a one-sample t-test. *p <0.05, **p <0.01, ***p <0.001.

Under basal conditions, inactive PBMCs did not affect HCC viability up to 24 h (**Figure 7A**), even in the presence of durvalumab (**Supplementary Figure 7A**), consistent with minimal PD-1 expression on resting CD8^+^ T cells (**Figures 7B, C**), thereby limiting their cytotoxic potential. In contrast, CD3/CD28-mediated activation induced robust T-cell activation, with a 28-fold increase in CD69 and a 9-fold increase in CD137 expression at 24 h (**Figures 7D, E**). PD-1 expression was also upregulated at both the transcript (**Supplementary Figure 7B**) and protein levels, increasing from 1-3% of PD-1+ cells in inactive CD8^+^ T cells to 13–14% following activation (**Figure 7B**), peaking at 24 h before slightly declining (**Figure 7C**).

Once activated, PBMCs induced a time- and dose-dependent cytotoxic response against HCC cells. JHH6 cells were highly sensitive, with less than 50% viability remaining at E:T ratios of 1:1 and 1:2 by 40 h and 70 h, respectively, and >90% cell death by 72 h at the higher ratio (**Figure 7F**). In contrast, HuH7 cells exhibited markedly delayed killing kinetics. At a 1:1 ratio, viability declined by ~60% at 96 h (kill time 50 [KT_50_] = 77 h), while at 1:2 only a ~28% reduction was observed even at 120 h (**Figure 7G**). These findings were corroborated by Annexin V/PI staining, which revealed an early increase in apoptotic HCC cells following PBMC co-culture, with a 1.8-fold increase at 4 h and a 4.7-fold increase at 24 h (p < 0.001; **Supplementary Figure 7C**). Consistently, MTT assays showed a 32% and 71% reduction in JHH6 viability at 48 h and 72 h (p = 0.005), respectively, compared with only a 26% reduction in HuH7 cells at 72 h (**Supplementary Figure 7D**).

Together, these data demonstrate that S1-type JHH6 cells, despite their higher PD-L1 expression, are highly susceptible to CD8^+^-mediated cytotoxicity following checkpoint activation, whereas S2-type HuH7 cells display intrinsic immune resistance. This supports the concept that elevated, stable PD-L1 reflects active immune engagement and a therapeutically targetable axis, while in S2 contexts, dynamic PD-L1 turnover and reliance on *de novo* synthesis limit responsiveness to PD-1/PD-L1 blockade alone, providing a mechanistic rationale for differential clinical outcomes and the need for combinatorial strategies.

### 3.9 Anti–PD-L1 antibodies rapidly saturate surface PD-L1 and reveal stable responder and non-responder host phenotypes

**Figure 8:**
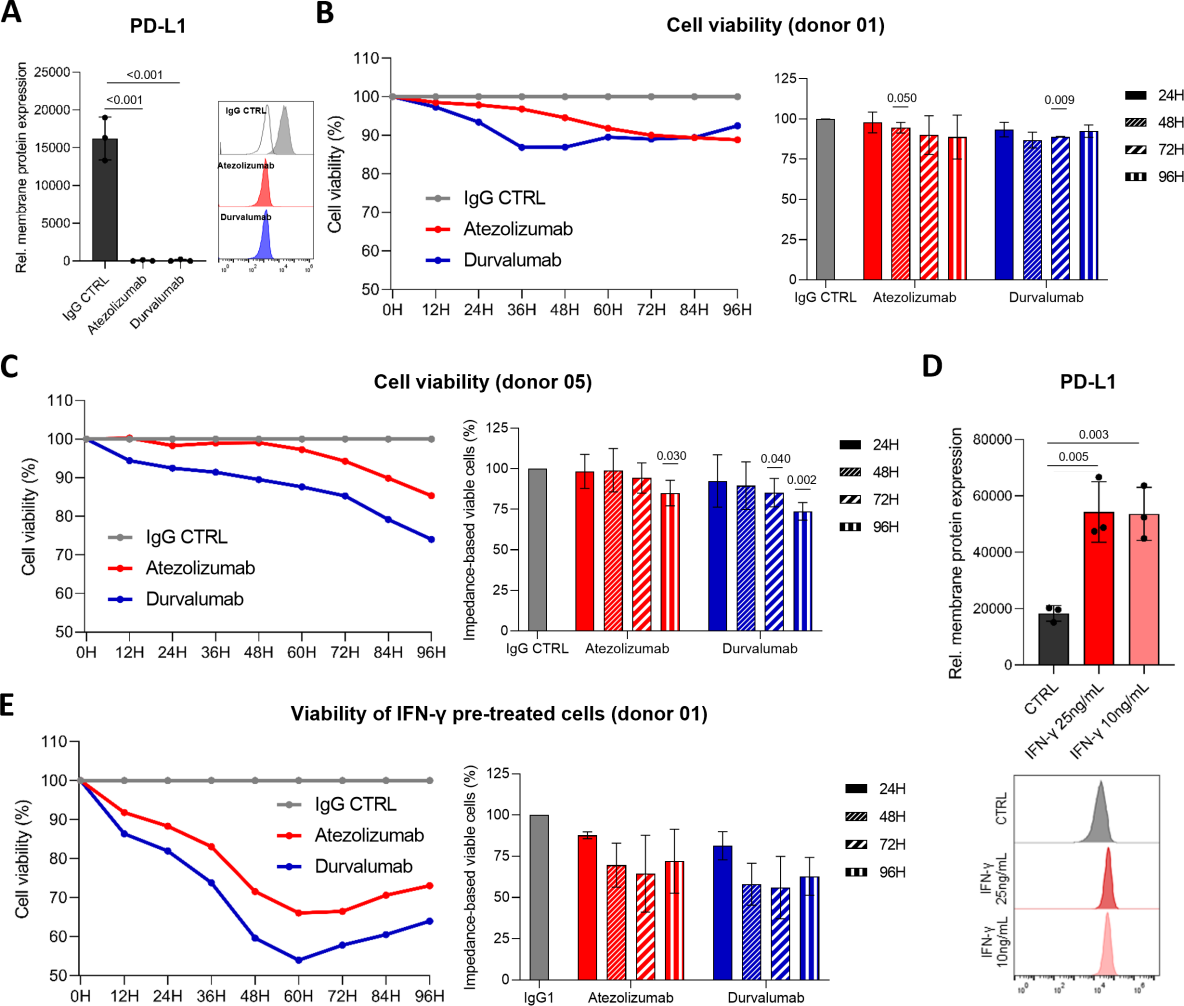
Impact of immune checkpoint blockade on HCC cell viability. **(A)** Representative PD-L1 membrane staining in JHH6 cells following treatment with atezolizumab or durvalumab (10 µg/mL; n = 3). Statistical significance was evaluated using an unpaired t-test. **(B, C)** Time-course, impedance-based viability of JHH6 cells co-cultured with PBMCs at an E:T ratio of 1:2, using PBMCs from donor 01 (n = 3) **(B)** and donor 05 (n = 4) **(C)**. Statistical significance was evaluated using a one-sample t-test. **(D)** PD-L1 membrane expression in JHH6 cells following 24-hour IFN-γ treatment (10 or 25 ng/mL; n = 3). Statistical significance was evaluated using an unpaired t-test. **(E)** Time-course, impedance-based viability analysis of JHH6 cells pretreated with IFN-γ (10 ng/mL for 24 h) and subsequently co-cultured with PBMCs from donor 01 (n = 2).

Both atezolizumab and durvalumab efficiently bound surface PD-L1 within 1 hour, confirming complete target engagement (**Figure 8A**), which remained stable over time (**Supplementary Figure 8A**), and did not alter PD-L1 transcriptional levels (**Supplementary Figure 8B**). To assess inter-individual variability in immunotherapy responsiveness, we performed co-culture experiments using PBMCs from multiple healthy donors. Notably, repeated sampling from the same donor, spaced by at least one week, yielded highly reproducible responses, indicating that the impact of PD-L1 blockade on HCC cell viability represents a stable, donor-intrinsic feature (**Supplementary Figure 8C**).

Across donors, heterogeneous responses to PD-L1 blockade were observed. While three donors exhibited a clear enhancement of CD8^+^ T cell–mediated cytotoxicity upon PD-L1 blockade, one donor (donor 03) showed no improvement in HCC cell killing relative to control (**Supplementary Figure 8C**), thereby defining stable responder and non-responder phenotypes. Among responders, donor 05 displayed the strongest and most consistent effect, with comparable efficacy across independent sampling time points (**Supplementary Figure 8C**). In this donor, enhanced tumor cell killing became most evident after 60–72 h of co-culture (**Supplementary Figure 8C**), a timeframe consistent with the membrane PD-L1 half-life measured in JHH6 cells (**Figures 3D, E, 3H**).

In JHH6 co-cultures, PBMCs from donor 01 induced a progressive reduction in tumor cell viability upon PD-L1 blockade, achieving an additional ~10% killing relative to isotype control at 72 h before reaching a plateau (**Figure 8B**). In line with these kinetics, neither atezolizumab nor durvalumab increased apoptosis at 24 h compared with control conditions (**Supplementary Figure 8D**). PBMCs from donor 05 exhibited a continuous decline in JHH6 viability, with a marked change in slope around 60–72 h (**Figure 8C**). Among PD-L1 blockade, durvalumab produced a stronger enhancement of cytotoxicity than atezolizumab, reducing JHH6 viability by 26% at 96 h relative to isotype control (p = 0.003), compared with a 15% reduction for atezolizumab (p = 0.020; **Figure 8C**).

These findings indicate that host immune competence represents a critical determinant of response to PD-L1 blockade, besides tumor PD-L1 expression or Hoshida subtype. This donor-dependent variability highlights the importance of systemic immune fitness in shaping both the magnitude and timing of immunotherapy responses.

### 3.10 IFN-γ enhances PD-L1 expression while restoring immunotherapy responsiveness

IFN-γ is a well-established inducer of PD-L1 expression, exerting a dual role by promoting antitumor immunity while simultaneously activating adaptive immune resistance mechanisms. To investigate how IFN-γ modulates PD-L1 regulation and immunotherapy response in HCC, S1 and S2 cells were treated with IFN-γ prior to co-culture experiments.

Treatment with 10 or 25 ng/mL IFN-γ induced a rapid and coordinated increase in all PD-L1 glycoforms, including both cytoplasmic and membrane-associated species, within 24 h (**Supplementary Figure 9A**). This resulted in an approximately 3-fold increase in PD-L1 protein levels in JHH6 cells (**Figure 8D**) and a pronounced 54-fold induction in HuH7 cells (**Supplementary Figure 9B**), accompanied by a parallel rise in *PD-L1* mRNA expression in both lines (**Supplementary Figure 9C**). Beyond activating the PD-L1 adaptive resistance axis, IFN-γ pre-treatment markedly enhanced the responsiveness of donors previously showing limited responses to PD-L1 blockade.

When IFN-γ–pretreated JHH6 cells were co-cultured with activated PBMCs from donor 01, immune checkpoint inhibition induced an earlier and more pronounced reduction in tumor cell viability compared with isotype control (24 h: −12% to −19%; 48 h: −30% to −42%), with maximal effects observed at 60 h (atezolizumab: -34%; durvalumab: -46%; **Figure 8E**). In contrast, in the absence of IFN-γ pretreatment, only a modest reduction in viability was observed (−10%, **Figure 8B**). This apparent paradox is consistent with emerging evidence that not only induces PD-L1 expression but also primes the tumor–immune interface by enhancing antigen presentation and chemokine-driven effector recruitment (39, 40). In this setting, the immuno-stimulatory effects outweigh PD-L1–mediated adaptive resistance, thereby increasing the efficacy of PD-L1 blockade.

These results highlight the central role of PD-L1 expression dynamics in shaping immune responsiveness and suggest that elevated PD-L1 can enhance ICI-mediated cytotoxicity, likely by increasing target availability and promoting immune activation.

## 4 Discussion

A central challenge in cancer immunotherapy is understanding why only a subset of HCC patients benefit from PD-1/PD-L1 blockade. Although PD-L1 has been widely explored as a biomarker, its predictive value in HCC remains inconsistent, likely due to profound intratumoral heterogeneity, multiple biochemical forms of PD-L1, and patient-specific immune competence. By integrating patient samples, an HBV-driven mouse model, and mechanistic *in vitro* systems, our study demonstrates that immunotherapeutic responsiveness in HCC is determined by the interplay of molecular subtype–specific PD-L1 regulation, tumor-intrinsic biochemical properties of PD-L1, and host immune cytotoxic potential. Thus, PD-L1 molecular diversity must be interpreted within the broader immunological landscape to understand clinical responses to immune checkpoint blockade.

Across public datasets and our cohort, *PD-L1* mRNA levels were largely unchanged between tumors and paired non-tumoral liver, indicating that transcriptional induction is not the primary driver of PD-L1 regulation. In the HBV-induced mouse model, early neoplastic lesions exhibited PD-L1 expression comparable to that of the adjacent diseased liver, although both were lower than in healthy controls, underscoring that chronic liver disease establishes the baseline PD-L1 landscape. In contrast, protein-level analyses revealed a significant increase in tumoral PD-L1 in human HCC, with upregulation observed in 61% of cases. Viral hepatitis further amplified PD-L1 expression, and tumor-to-adjacent PD-L1 ratios inversely correlated with BMI, suggesting that inflammatory immune pressure—particularly in virally driven contexts—contributes to PD-L1 induction. Mechanistically, HBV has been shown to upregulate PD-L1 by blocking poly(ADP-ribose) polymerase 1 (PARP1) nuclear translocation through direct interaction with the HBV DNA polymerase (41), while the hepatitis C virus (HCV) core protein induces PD-L1 expression on Kupffer cells and monocytes *via* toll-like receptor 2 (TLR2) and phosphoinositide 3-kinase (PI3K)-dependent pathways (42, 43), and the HCV F protein promotes PD-1 expression on CD8^+^ and CD4^+^ T cells, contributing to T-cell exhaustion during chronic infection (29). Consistently, we confirmed that IFN-γ is a potent inducer of PD-L1 (44, 45), and elevated PD-L1 expression in non-tumoral liver was associated with increased recurrence, highlighting the immunosuppressive microenvironment within the residual liver as a potential driver of relapse.

Biochemical profiling revealed a complex PD-L1 glycoprofile. Beyond the canonical ~50 kDa glycosylated membrane form, tumors exhibited multiple intermediate species (~45, ~39, ~38, and ~35 kDa), reflecting altered maturation and trafficking dynamics within the tumor microenvironment (10, 46, 47). The prevalence of glycosylated PD-L1 in early-stage tumors suggests active immune evasion occurs early in hepatocarcinogenesis and supports the rationale for preoperative immune checkpoint blockade (48, 49). Consistently, clinical studies with cabozantinib–nivolumab, camrelizumab–apatinib, or nivolumab ± ipilimumab have reported substantial pathological responses, converting borderline cases to resectable disease and improving DFS (50–52). These clinical responses are highly variable across patients, and our data suggest that differences in PD-L1 biochemical composition and stability may contribute to this heterogeneity beyond expression levels alone.

In cell models, glycosylated PD-L1 demonstrated remarkable stability, particularly in S1/CL3 tumor subclasses, whereas less glycosylated intermediates were rapidly degraded. Because therapeutic antibodies rely on glycan-dependent epitope recognition, PD-L1 glycosylation carries important implications for clinical efficacy: atezolizumab binding is partially glycoform dependent and diminishes when glycosylation is altered, whereas durvalumab, characterized by higher affinity and reduced glycoform dependence, remains largely unaffected (10, 53). Consistent with this biochemical distinction, our functional assays demonstrated superior activity of durvalumab across *in vitro* co-culture systems. These findings raise the possibility that PD-L1 glycosylation status may influence not only biomarker detection but also the relative efficacy of different PD-L1–targeting antibodies in clinical settings.

Current PD-L1 evaluation relies primarily on static IHC measurements (54), which do not capture the biochemical and regulatory heterogeneity highlighted in our study. Glycosylation modulates epitope accessibility, and antibodies targeting different domains—cytoplasmic, membrane-associated, or extracellular—can yield divergent results (32). Notably, deglycosylation markedly enhances PD-L1 detection (55), underscoring the need for refined, glycoform-aware PD-L1 assays in HCC to improve biomarker accuracy and patient stratification. From a clinical perspective, these observations argue against the use of PD-L1 expression as a standalone biomarker and support the development of composite biomarkers integrating PD-L1 biochemical features, molecular subclassification, and immune contexture.

Mapping hepatoma cell lines to Hoshida/Caruso subclasses, we found that S1/CL3 cells displayed high PD-L1 abundance, consistent with primary S1 tumors characterized by immune infiltration (14) and activation of the TGF-β pathway (13, 16), which has been implicated in PD-L1 induction (56). S1/CL3 cells exhibited robust post-translational stabilization, maintaining membrane-associated PD-L1 even when degradative pathways were inhibited, and rapidly accumulating immature PD-L1 intermediates under proteotoxic stress. Previous studies in breast cancer have shown that glycosylated PD-L1 has an approximately fourfold longer half-life than non-glycosylated forms, which are degraded within ~4 hours (9). Our data extend these observations by demonstrating a markedly prolonged PD-L1 half-life in S1-JHH6 cells. Consistent with this, the extent of PD-L1 glycosylation correlated with both baseline PD-L1 abundance and protein stability across molecularly defined HCC subtypes (10, 46). Paradoxically, despite their high PD-L1 expression, S1 cells were more susceptible to CD8^+^ T-cell–mediated killing, indicating that elevated PD-L1 reflects active immune engagement rather than intrinsic immune resistance. This supports the concept that elevated, stable PD-L1 reflects active immune engagement and a therapeutically targetable axis.

Host immune competence emerged as a critical determinant of therapeutic efficacy. PBMCs from different healthy donors exhibited reproducible cytotoxic phenotypes across independent sampling times, ranging from stable non-responder profiles to highly effective responder phenotypes, potentially reflecting differences in cytotoxic effector subsets (57, 58). The magnitude and timing of tumor killing following PD-L1 blockade depended strongly on donor-derived immune competence and aligned with PD-L1 membrane stability in S1 cells.

Remarkably, IFN-γ pre-treatment enhanced immunotherapy responsiveness in previously less responsive donors by boosting antigen presentation, effector recruitment, and the expression of multiple immune checkpoints through activation of the janus kinase (JAK)/signal transducer and activator of transcription 1 (STAT1) pathway and downstream signaling cascades, including nuclear factor kappa-light-chain-enhancer of activated B cells (NF-κB), PI3K/AKT, and metabolic reprogramming (59–61). These features define an immune-hot phenotype characterized by increased T-cell infiltration and greater sensitivity to ICIs (62), whereas immune-cold tumors lack these features, accounting for their limited responsiveness to ICIs as monotherapy (63). These findings reconcile the apparent paradox of IFN-γ–driven PD-L1 upregulation: rather than representing a purely immunosuppressive mechanism, PD-L1 induction in this context may serve as a surrogate marker of an active, inflamed tumor microenvironment that remains responsive to immune checkpoint blockade.

Importantly, our data support a unifying framework in which the clinical efficacy of PD-1/PD-L1 blockade is determined by the convergence of PD-L1 molecular state and the underlying immune landscape. Tumors with highly glycosylated, membrane-stable PD-L1 (S1-like) are associated with inflamed microenvironments and dependence on PD-1/PD-L1 signaling, rendering them more likely to respond to therapy. In contrast, S2/CL1 cells displayed low PD-L1 expression, limited membrane localization, and a predominant reliance on *de novo* synthesis rather than post-translational stabilization. This dynamic regulation of PD-L1 likely limits the effectiveness of PD-L1 blockade alone. These features provide a mechanistic basis for the immune-cold phenotype and resistance to PD-L1–directed therapies observed in S2 tumors, and support the rationale for combinatorial strategies aimed at modulating PD-L1 dynamics in addition to immune checkpoint inhibition. Consistent with clinical observations, only a subset of HCC patients derives durable benefit from immune checkpoint inhibitors, with response rates of approximately 15–30%, and our findings suggest that this variability reflects the combined influence of PD-L1 biochemical diversity, tumor subtype, immune contexture, and host immune competence rather than expression levels alone. These differences are likely to impact not only response rates but also progression-free survival, OS, and recurrence risk, particularly in the perioperative setting.

From a clinical perspective, these results support a shift toward composite biomarker strategies integrating PD-L1 biochemical features, molecular classification, and immune profiling to improve patient stratification and better predict clinical outcomes. Therapeutically, this framework suggests that immune-inflamed tumors with stable PD-L1 may benefit from PD-1/PD-L1 blockade–based approaches, whereas immune-cold tumors may require combination strategies targeting alternative checkpoints or interventions aimed at enhancing tumor immunogenicity. Future studies should focus on prospective validation of these parameters and on the development of clinically applicable assays capturing PD-L1 biochemical states to refine treatment selection and improve patient outcomes. 

This study has several limitations. PBMC–HCC co-culture systems provide mechanistic insights but cannot fully recapitulate the complexity of the tumor microenvironment. Further validation in patient-derived and *in vivo* models is warranted. In addition, although we focused on proteasomal PD-L1 turnover, other regulatory mechanisms—including autophagy, lysosomal degradation, hypoxia, and inflammatory mediators—are likely to contribute to PD-L1 regulation and merit further investigation.

## 5 Conclusions

PD-L1 biology in HCC extends beyond expression levels to encompass subtype-specific differences in glycosylation, stability, and turnover, limiting the predictive value of PD-L1 alone for immune-mediated cytotoxicity, particularly in tumors engaging alternative inhibitory pathways. In the context of clinically approved immune checkpoint inhibitors, we show that PD-L1 targeting induces immune-mediated killing with marked donor-specific variability and only in a subset of tumor cells, consistent with the limited fraction of HCC cases expressing PD-L1 on malignant cells (26). Heterogeneity across molecular subtypes, antibody modalities, and donor immune profiles highlights fundamental differences between immune-hot and immune-cold tumors and underscores the need for combinatorial strategies to overcome resistance (64).

Together, these findings support personalized immunotherapy approaches integrating PD-L1 biochemical stability, molecular subclassification, and functional immune profiling to refine patient stratification and optimize perioperative and early-stage HCC treatment.

## Data Availability

The raw data supporting the conclusions of this article will be made available by the authors, without undue reservation, at: https://www.fegato.it/grisetti_front_imm/.

## Supplementary material

The Supplementary Material for this article can be found online at: https://www.frontiersin.org/articles/10.3389/fimmu.2026.1822947/full#supplementary-material and https://www.fegato.it/grisetti_front_imm/.
