# Supplementary material for: Subtype-dependent PD-L1 stability and immune context shape immunotherapy response in hepatocellular carcinoma
Source: Front Immunol. 2026 May 1;17:1822947. doi: 10.3389/fimmu.2026.1822947 (PMC13176238; doi:10.3389/fimmu.2026.1822947)
Supplement: Supplementary file 1 [file DataSheet1.pdf]

Supplementary Figures

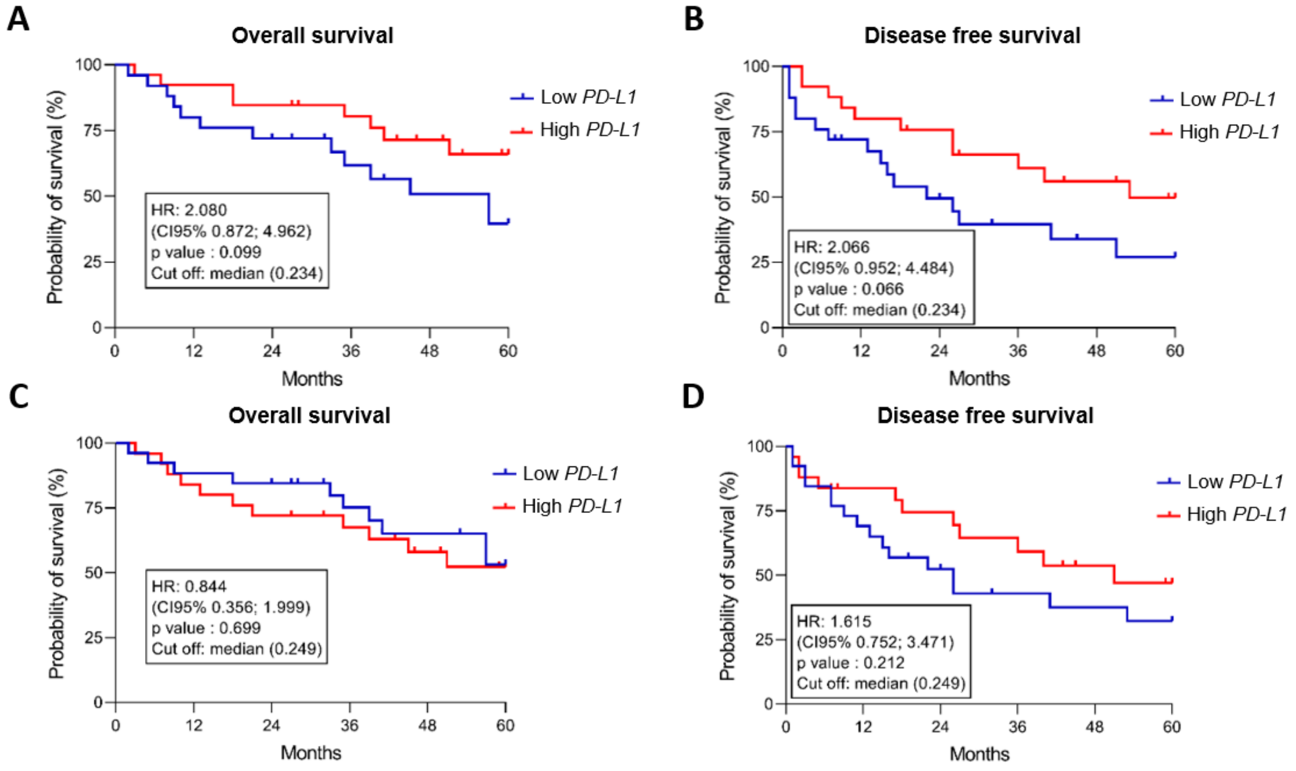

Supplementary Figure 1. *PD-L1* mRNA and patient stratification. OS (**A**) and DFS (**B**) curves stratified by *PD-L1* tumoral expression (cut-off: median). OS (**C**) and DFS (**D**) curves stratified by *PD-L1* expression in paired adjacent non-tumoral tissues (cut-off: median).

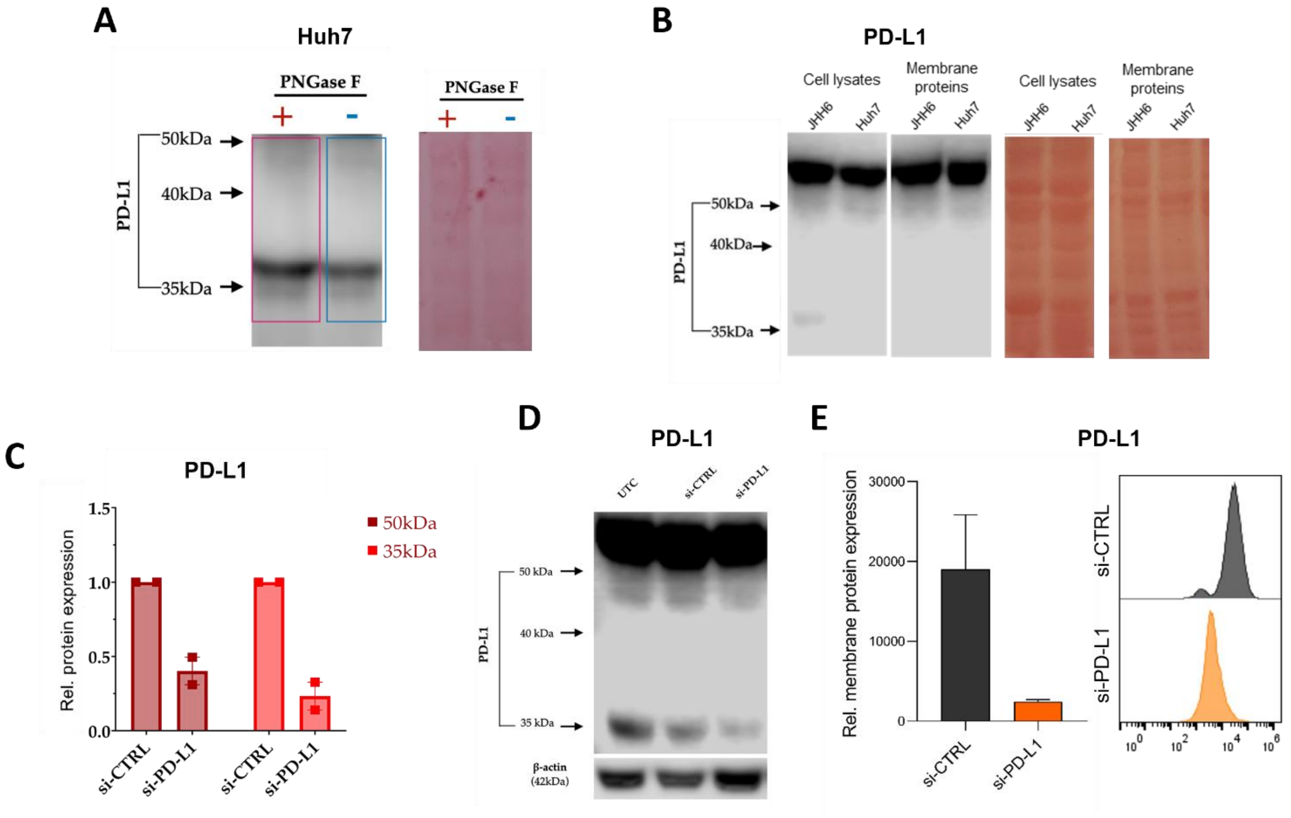

Supplementary Figure 2. PD-L1 displays multiple biochemical species in HCC cells with distinct glycosylation, localization, and proteasome regulation. (**A**) PNGase F treatment in HuH7 cells demonstrating PD-L1 de-glycosylation (red box). Ponceau Red staining is shown as a loading control. (**B**) Comparison of PD-L1 band distribution in crude membrane fractions *vs.* whole-cell lysates from JHH6 and HuH7 cells; the ~50 kDa band corresponds to membrane-associated PD-L1. Ponceau Red staining is shown as a loading control. (**C**, **D**) Relative expression of ~33–37 kDa and ~50 kDa PD-L1 species (**C**) and representative immunoblot (**D**) following 72-hour siRNA-mediated PD-L1 silencing in HuH7 cells (n = 2). UTC, untreated control. (**E**) Relative PD-L1 membrane expression following PD-L1 silencing in JHH6 cells, assessed by flow cytometry (n = 2).

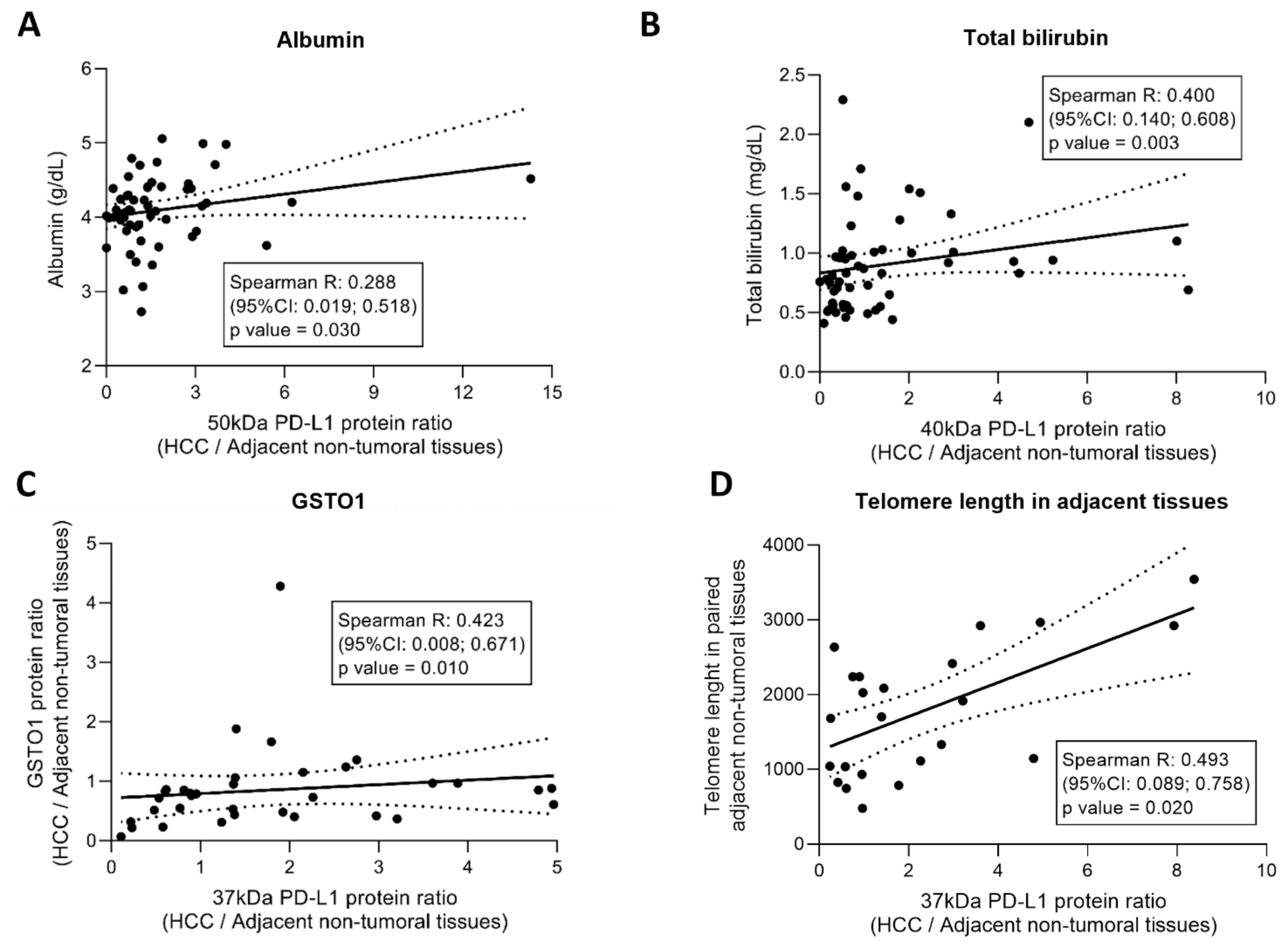

Supplementary Figure 3. Correlation between PD-L1 biochemical species and clinical or molecular features. (**A**) Correlation between 50 kDa PD-L1 tumor-to-adjacent ratios and serum albumin levels. (**B**) Correlation between 40 kDa PD-L1 tumor-to-adjacent ratios and total bilirubin levels. (**C**, **D**) Correlation between 37 kDa PD-L1 tumor-to-adjacent ratios and GSTO1 tumor-to-adjacent ratios (**C**) and telomere length in adjacent non-tumoral tissues (**D**).

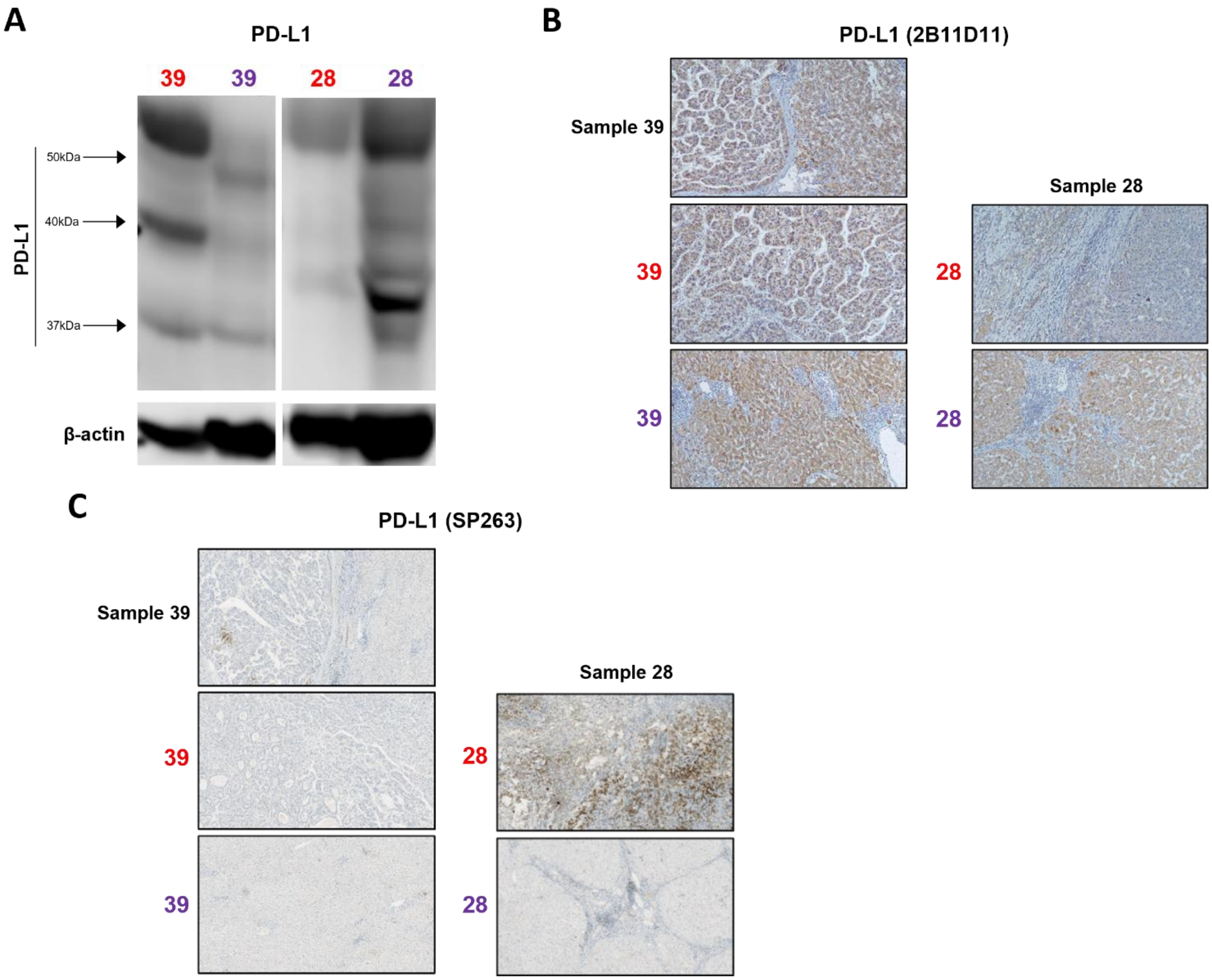

Supplementary Figure 4. PD-L1 IHC in representative HCC patients. (**A**) Immunoblots resolving PD-L1 bands in sample 39 (increased tumor-to-adjacent PD-L1 ratio) and sample 28 (decreased tumor-to-adjacent PD-L1 ratio). Red: tumor; purple: adjacent non-tumoral tissues. (**B**, **C**) PD-L1 IHC staining in samples 39 and 28 using the 2B11D11 (**B**) and SP263 (**C**) antibody clones. Top panels show the tumor–adjacent interface; middle panels show tumor tissue; bottom panels show adjacent non-tumoral tissue. Magnification: 10×.

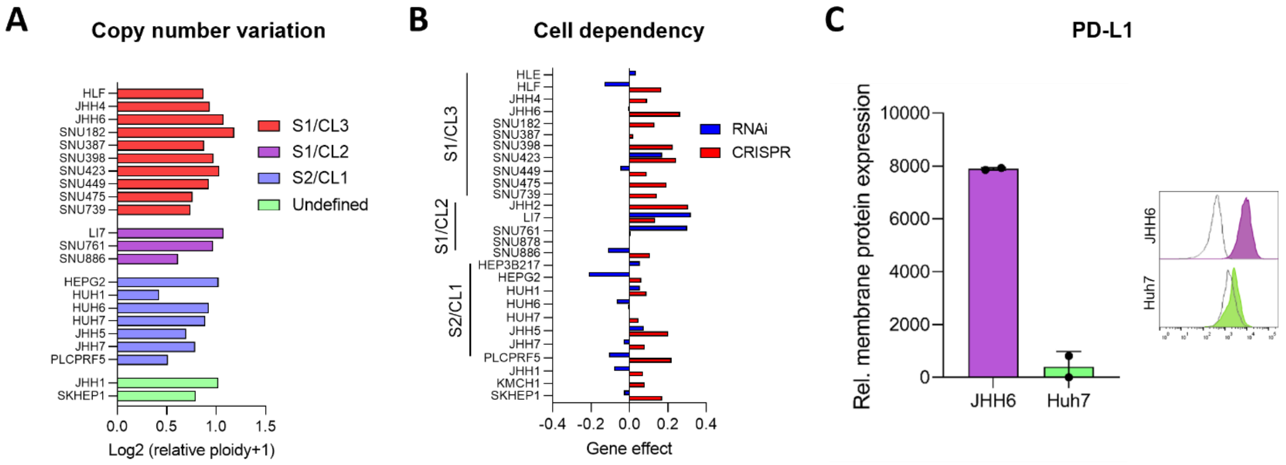

Supplementary Figure 5. PD-L1 expression and dependency in HCC cell lines. (**A**) PD-L1 copy number variation in liver cancer cell lines (DepMap). (**B**) PD-L1 dependency scores derived from RNAi and CRISPR screening datasets (DepMap). (**C**) Relative membrane-associated PD-L1 expression assessed by flow cytometry (n = 2), normalized to isotype control (grey).

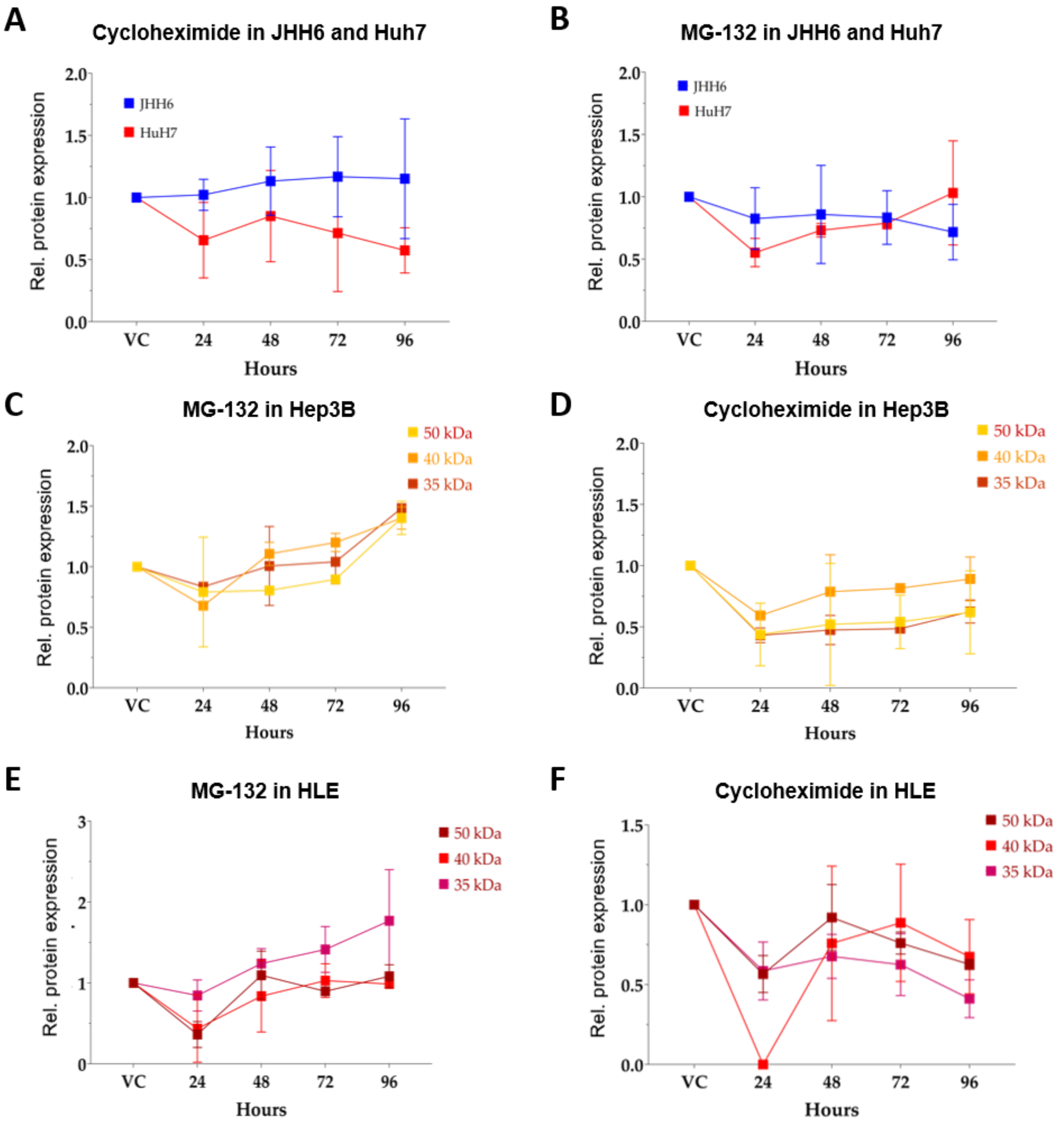

Supplementary Figure 6. PD-L1 protein expression dynamics in additional HCC lines. (**A**, **B**) Relative changes in total PD-L1 expression following CHX chase (**A**) or MG-132–mediated proteasome inhibition (**B**) (24–96 h) in JHH6 and HuH7 cells. VC, vehicle control. (**C**, **D**) Relative PD-L1 expression following MG-132 treatment (**C**) or CHX chase (**D**) (24–96 h) in Hep3B cells (n = 3). VC, vehicle control. (**E**, **F**) Relative PD-L1 expression following MG-132 treatment (**E**) or CHX chase (**F**) (24–96 h) in HLE cells (n = 3). VC, vehicle control.

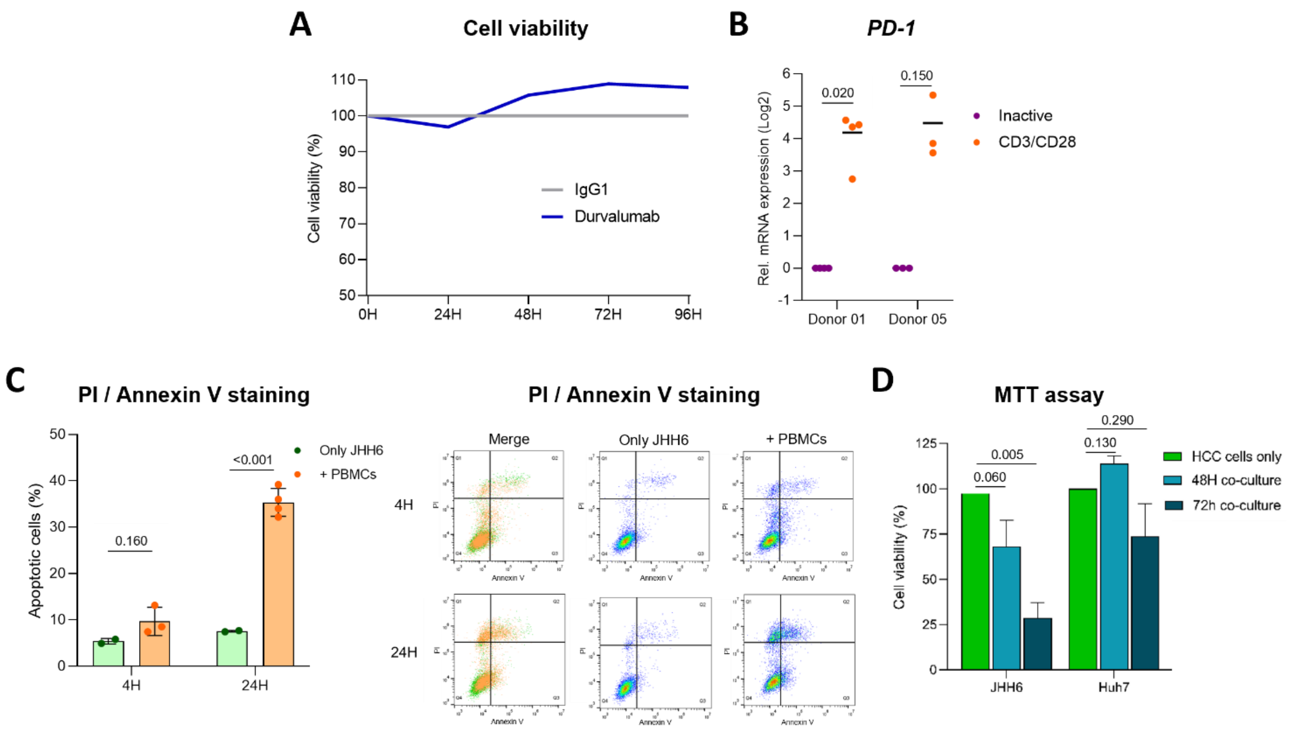

Supplementary Figure 7. Impact of PBMC activation on HCC cell viability and PD-1 expression. (**A**) Impedance-based viability of JHH6 cells following co-culture with non-activated PBMCs (donor 01). (**B**) Relative *PD-1* mRNA expression in non-activated *vs.* CD3/CD28-activated $CD8^+$ T cells, assessed by qRT-PCR. Statistical significance was evaluated using a one-sample t-test. (**C**) Annexin V/PI apoptosis analysis in JHH6 cells after 4 h (E:T = 2:1, n = 3) or 24 h (E:T = 1:1, n = 4) of co-culture. Apoptotic cells are defined as those in quadrants Q1, Q2, and Q3. Statistical significance was evaluated using an unpaired t-test. (**D**) MTT-based viability of JHH6 (n = 3) and HuH7 (n = 2) cells at 48 h and 72 h of co-culture (E:T = 1:1). Statistical significance was evaluated using a one-sample t-test.

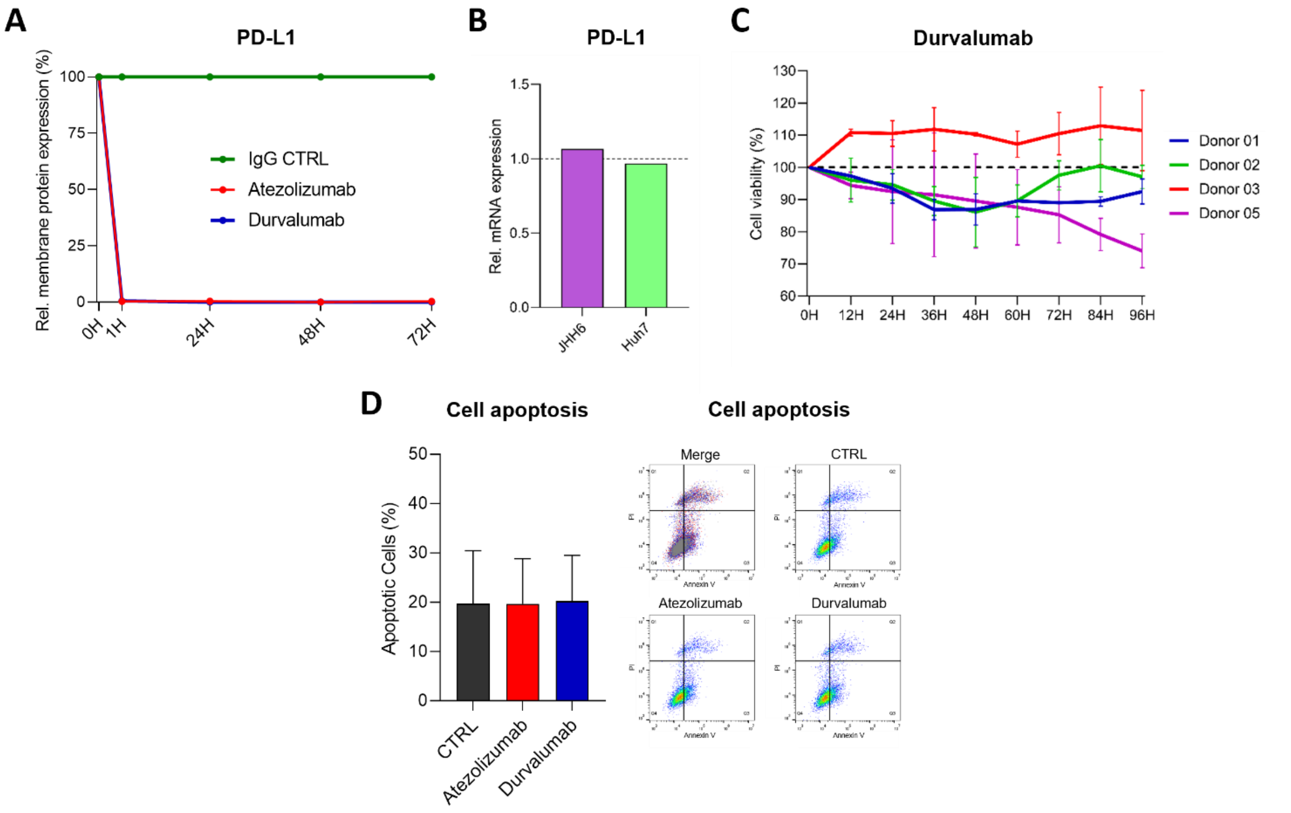

Supplementary Figure 8. Effects of immune checkpoint inhibitors on PD-L1 expression and HCC cell survival. (**A**) PD-L1 membrane staining in JHH6 cells following treatment with atezolizumab or durvalumab (10 µg/mL; n = 1). (**B**) Relative *PD-L1* mRNA expression in JHH6 cells after 24 h of atezolizumab treatment (10 µg/mL; n = 1). (**C**) Time-course impedance-based viability of JHH6 cells treated with durvalumab and co-cultured with PBMCs from donors 01, 02, 03, and 05 at an E:T ratio of 1:2 (n ≥ 2). (**D**) Annexin V/PI apoptosis analysis in JHH6 cells after 24 h of co-culture with PBMCs (donor 01; E:T = 1:1; n = 2). Apoptotic cells are defined as those in quadrants Q1, Q2, and Q3.

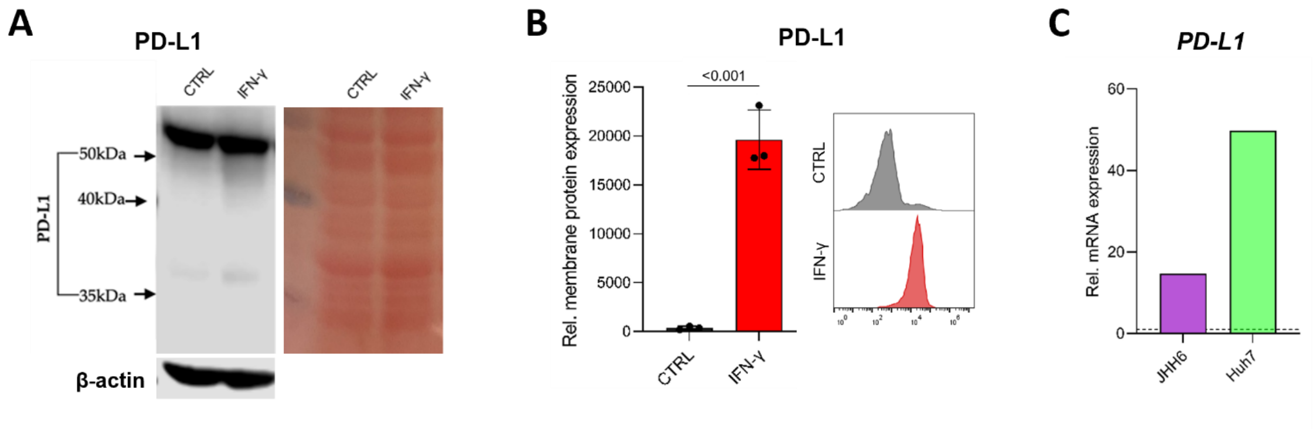

Supplementary Figure 9. IFN-γ-mediated regulation of PD-L1 expression. (**A**) Representative immunoblot showing PD-L1 glycoforms (~33–37 kDa, ~39–45 kDa, and ~50 kDa) following 24 h of IFN-γ stimulation (10 ng/mL; n = 1). Ponceau Red staining is shown as a loading control. (**B**) PD-L1 surface expression in HuH7 cells following 24 h of IFN-γ treatment (25 ng/mL; n = 3). Statistical significance was evaluated using an unpaired t-test. (**C**) Relative *PD-L1* mRNA expression in JHH6 and HuH7 cells after 24 h of IFN-γ stimulation (25 ng/mL; n = 3).
